# A Complete Solution for Dissecting Pure Main and Epistatic Effects of QTL in Triple Testcross Design

**DOI:** 10.1371/journal.pone.0024575

**Published:** 2011-09-19

**Authors:** Xiao-Hong He, Yuan-Ming Zhang

**Affiliations:** Section on Statistical Genomics, State Key Laboratory of Crop Genetics and Germplasm Enhancement/Chinese National Center for Soybean Improvement, College of Agriculture, Nanjing Agricultural University, Nanjing, Jiangsu, China; University of Michigan, United States of America

## Abstract

Epistasis plays an important role in genetics, evolution and crop breeding. To detect the epistasis, triple test cross (TTC) design had been developed several decades ago. Classical procedures for the TTC design use only linear transformations Z_1_, Z_2_ and Z_3_, calculated from the TTC family means of quantitative trait, to infer the nature of the collective additive, dominance and epistatic effects of all the genes. Although several quantitative trait loci (QTL) mapping approaches in the TTC design have been developed, these approaches do not provide a complete solution for dissecting pure main and epistatic effects. In this study, therefore, we developed a two-step approach to estimate all pure main and epistatic effects in the F_2_-based TTC design under the F_2_ and F_∞_ metric models. In the first step, with Z_1_ and Z_2_ the augmented main and epistatic effects in the full genetic model that simultaneously considered all putative QTL on the whole genome were estimated using empirical Bayes approach, and with Z_3_ three pure epistatic effects were obtained using two-dimensional genome scans. In the second step, the three pure epistatic effects obtained in the first step were integrated with the augmented epistatic and main effects for the further estimation of all other pure effects. A series of Monte Carlo simulation experiments has been carried out to confirm the proposed method. The results from simulation experiments show that: 1) the newly defined genetic parameters could be rightly identified with satisfactory statistical power and precision; 2) the F_2_-based TTC design was superior to the F_2_ and F_2:3_ designs; 3) with Z_1_ and Z_2_ the statistical powers for the detection of augmented epistatic effects were substantively affected by the signs of pure epistatic effects; and 4) with Z_3_ the estimation of pure epistatic effects required large sample size and family replication number. The extension of the proposed method in this study to other base populations was further discussed.

## Introduction

Epistasis, the interaction between genes, plays an important role in genetics, evolution and crop breeding. First, it is an important genetic component in the genetic architecture of complex traits [1], [2]. Next, it can lead to heterosis [3]–[7], which is very important in hybrid breeding. In addition, it is a driving force in evolution and plays a central role in founder effect models of speciation [1], [8], [9]. Over the past several decades, many attempts have been made to detect the epistasis. One important attempt was triple test cross (TTC) design developed by Kearsey and Jinks [10], which is a powerful breeding design as well. Therefore, the great importance associated with the epistasis necessitates an in-depth study of the TTC design.

The TTC design is to cross the *i*th individual (*i* = 1,2,…*n*) of an F_2_ population (or backcross, recombinant inbred lines (RIL) and near isogenic lines (NIL)) to the same three testers, the two inbred lines (P_1_ and P_2_) and their F_1_, to produce 3*n* families. The design is considered the most efficient model as it provides not only a precise test for epistasis, but also unbiased estimates of additive and dominance components if epistasis is absent [10]. In early studies, only the phenotypic data of quantitative traits were used in the TTC to infer the nature of the additive, dominance and epistatic effects of polygenes using classical generation mean [11]–[13] and variance component analysis [10], [12], [14]–[17]. However, these conventional biometrical genetic procedures deal only with the collective effects of all the polygenes [6], [7], [11], [12]. The introduction of molecular markers has facilitated the mapping of quantitative trait loci (QTL) in numerous species, and substantial progress has been achieved in the detection of individual QTL and their interaction in the RIL- and NIL-based TTC designs.

In the RIL-based TTC designs, Kearsey et al. [12] employed the marker difference regression of Kearsey and Hyne [18] to detect QTL for 22 quantitative traits in *Arabidopsis thaliana*. Frascaroli et al. [16] used composite interval mapping [19] to identify main-effect QTL and the mixed linear model approach [20] to detect digenic epistatic QTL in the analyses of heterosis in maize. The method has been used to identify the main-effect QTL and digenic epistatic QTL underlying the heterosis of nine important agronomic and economic traits in rice by Li et al. [17]. However, the additive and dominant effects estimated from the above approaches are confounded with epistatic effect if epistasis is present. To overcome this issue, Melchinger et al. [21] derived quantitative genetic expectations of QTL main and interaction effects in the RIL-based TTC design. On their theoretical findings, using one-dimensional genome scans, we can estimate augmented additive and dominance effects [7] and QTL- by-genetic background interaction, whereas using two-way ANOVA between all pairs of marker loci, we can estimate additive-by-additive (*aa*) and dominance-by-dominance (*dd*) interactions. Kusterer et al. [22] applied the novel approaches of Melchinger et al. [7], [21] to detect QTL for heterosis of biomass-related traits in *Arabidopsis*. In the above studies, only one variable was involved at one time. To increase the power of QTL detection, Kusterer et al. [22] adopted multi-variable joint analysis [23], as proposed by Melchinger et al. [7] for QTL mapping in the NCIII design.

In the NIL-based TTC design, Melchinger et al. [21] used two QTL mapping methods to study heterosis in *Arabidopsis*. In the generation means approach, additive, dominance and QTL × genetic background epistasis effects were tested and estimated, and the approach along with particular two-segment NILs was applied by Reif et al. [24] to map *aa* digenic interaction. In addition, Zhu and Zhang [25] derived formulae for calculating the statistical power in the detection of epistasis; and Wang et al. [26] used interval mapping [27] to detect QTL underlying endosperm traits and demonstrated that the TTC provided a reasonably precise and accurate estimation of QTL positions and effects, especially the two dominant effects, which perfectly overcomes the drawback of the F_2:3_ design.

In summary, two issues in the detection of QTL in the TTC need to be addressed. First, only a few studies are built on F_2_-based TTC [25], [26], whereas most are built on RIL [7], [12], [16], [17], [21], [22] and NIL [6], [24]. Second, additive and dominance effects were confounded with QTL-by-genetic background interaction [7], [21], [22] and only *aa* and *dd* digenic interactions were evaluated in the RIL-based TTC [16], [17], [21], [22].

The objective of this study was to estimate, in an unambiguous and unbiased manner, all the main and epistatic effects of QTL in the F_2_-based TTC design. A series of Monte Carlo simulation experiments was carried out to confirm the proposed approach. The extension of the new method to other base populations in the TTC was discussed as well.

## Methods

### Genetic design and data collection

An F_2_ population was derived from two inbred lines (P_1_ and P_2_) that differed significantly in the quantitative traits of interest and possessed abundant polymorphism molecular markers. A random sample of *n* F_2_ individuals (female parents) was backcrossed to three testers, the two parental lines and their F_1_, to produce 3*n* families (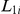, 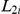 and 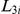). All of the 3*n* families, each with *m* replications, were planted. Molecular marker information was observed from all of the *n* F_2_ individuals, whereas quantitative traits were measured for all of the 3*nm* TTC progeny. The phenotypic observations were denoted by 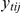, where 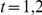 and 3 for 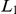, 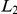 and 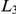; 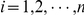 and 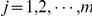. The family means were denoted by 
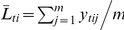
. Following Kearsey and Jinks [10] and Melchinger et al. [21], we performed three linear transformations: 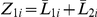, 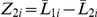 and 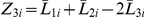. The association between 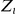 and the marker genotypes of the F_2_ plants were used to infer the genetic architecture of the trait.

### Genetic models for mapping QTL in the F_2_-based TTC design

The expected genetic values of 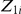, 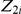 and 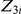 depended on the choice of the metric. Two main metrics, the F_2_ and F_∞_ metrics, were adopted for the populations derived from the cross between the two inbred lines [28]-[30]. The derivation of the expected genetic values of 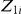, 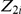 and 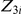 under both the F_2_ and the F_∞_ metric models was presented in Table S1, Table S2, Table S3, Table S4, Table S5, Table S6, and **Supporting Information S2**. The genetic effect symbols adopted in this study were referred to Kao and Zeng [28].

#### Statistical genetic models for mapping QTL under the F_2_ metric model

According to the expected genetic value of 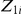 under the F_2_ metric model in Table S5, the phenotypic value of 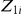 can be described as:
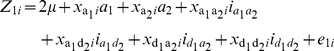
(1)where 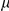 is the mean genotypic value of the F_2_ population; 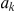 and 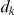 are additive and dominance effects of the *k*th QTL, respectively; 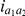, 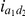, 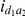 and 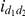 are additive-by-additive, additive-by-dominance, dominance-by-additive and dominance-by-dominance interactions between the 1st and 2nd QTL, respectively; 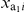, 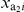, 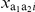, 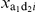, 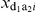 and 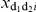 are dummy variables and determined by the genotype of the *i*th F_2_ plant (Table S5); and 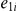 is the residual error with an 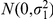 distribution. According to the results in Table S5, there are 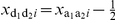, 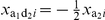 and 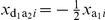. To solve the genetic parameters, model (1) must be reduced to:
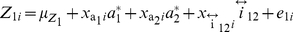
(2)where 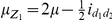, 
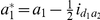
, 
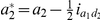
, 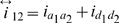 and 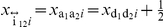.

If the quantitative trait was controlled by 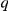 QTL, model (2) should be extended to:
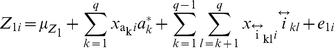
(3)where model mean 
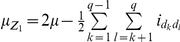
; 
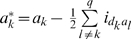
 is augmented additive effect of QTL 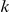; 
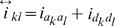
 is augmented epistatic effect between QTL 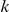 and 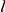; and 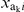 and 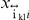 are determined by the genotypes of the *k*th and *l*th QTL (marker) of the *i*th F_2_ plant (Table 1). The coefficients for the genotype 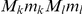 were integrated by the frequencies of 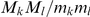 and 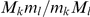. The augmented epistatic effects (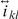) are ignored in Melchinger et al. [21], this may result in a bigger residual error and lower statistical power.

**Table 1:** Dummy variable values for genetic parameters in the genetic model of 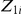, 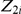 and 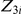 under various marker genotypes of F_2_ plant and the F_2_ and the F_∞_ metric models.

Marker genotype of F_2_ plant	F_2_ metric model															F_∞_ metric model		
	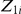													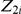	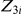	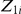	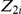	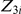
	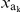	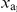	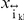	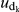	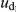	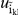	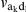	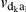	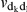	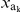	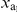	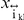	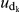	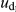	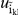	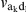	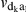	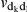
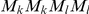	1	1	1	−1	−1	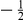	−*r*	−*r*	*r*	1	1	1	−1	−1	0	−*r*	−*r*	*r*
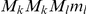	1	0	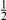	−1	0	0	0	−*r*	0	1	0	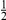	−1	0	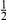	0	−*r*	0
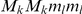	1	−1	0	−1	1	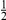	*R*	−*r*	−*r*	1	−1	0	−1	1	1	*R*	−*r*	−*r*
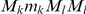	0	1	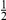	0	−1	0	−*r*	0	0	0	1	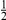	0	−1	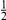	−*r*	0	0
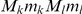	0	0	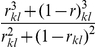	0	0	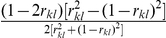	0	0	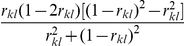	0	0	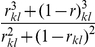	0	0	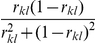	0	0	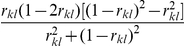
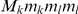	0	−1	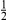	0	1	0	*r*	0	0	0	−1	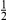	0	1	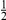	*R*	0	0
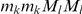	−1	1	0	1	−1	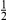	−*r*	*R*	−*r*	−1	1	0	1	−1	1	−*r*	*R*	−*r*
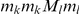	−1	0	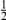	1	0	0	0	*R*	0	−1	0	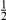	1	0	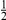	0	*R*	0
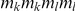	−1	−1	1	1	1	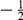	*r*	*r*	*r*	−1	−1	1	1	1	0	*r*	*r*	*r*

In the same way, the phenotypic value of 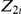 can be described as:
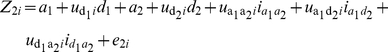
(4)where 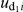, 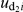, 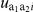, 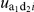 and 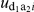 are determined by the genotype of the *i*th F_2_ plant (Table S5); and 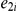 is the residual error with an 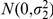 distribution. According to the results in Table S5, there are 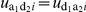 and 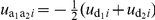. To solve the genetic parameters, model (4) must be reduced to:
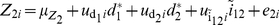
(5)where 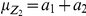, 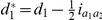, 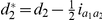, 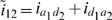 and 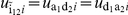.

If the quantitative trait was controlled by 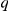 QTL, model (5) should be extended to:
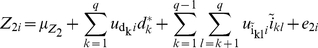
(6)where model mean 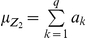; 
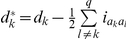
 is augmented dominance effect of QTL 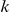; 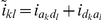 is augmented epistatic effect between QTL 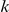 and 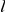; and dummy variables 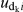 and 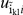 are determined by the genotypes of the *k*th and *l*th QTL of the *i*th F_2_ plant (Table 1). The augmented epistatic effects (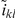) are overlooked in Melchinger et al. [21], this may result in a bigger residual error and lower statistical power.

Similarly, the phenotypic value of 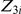 can be described as:
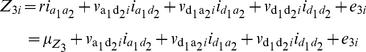
(7)where 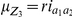; 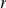 is the recombination fraction between two QTL under study; and dummy variables 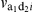, 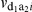 and 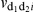 are determined by the genotype of the *i*th F_2_ plant (Table 1 and Table S5). Here pure *ad*, *da* and *dd* epistatic effects can be estimated with two-dimensional genome scans. This differs from that in Melchinger et al. [21], in which only *dd* epistasis is estimated with two-way ANOVA.

Models (3), (6) and (7) were working models for our QTL mapping approach in the F_2_-based TTC design. Here we proposed a two-step approach to obtain all the pure main and epistatic effects in the presence of epistasis. In the first step, model (3) can be used to estimate the augmented additive (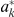) and epistatic (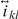) effects, model (6) can be used to estimate the augmented dominance (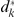) and epistatic (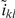) effects, and model (7) can be used to estimate three types of pure epistatic effects (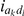, 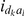 and 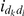). In the second step, all estimated epistatic effects in models (3), (6) and (7) were integrated for the estimation of all four types of the pure epistatic effects using 
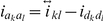
, 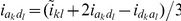 and 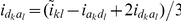. These pure epistatic effects further integrate with the estimates of both 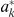 and 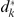 for the estimation of pure additive and dominance effects, using 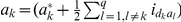 and 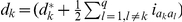. When epistasis is absent, pure additive (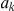) and dominance (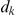) effects can be directly obtained from model (3) and model (6), respectively.

#### Genetic models for mapping QTL under the F_∞_ metric model

With 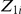, 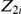 and 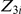 genetic models for mapping QTL under the F_∞_ metric model have the same forms as described in models (3), (6) and (7), respectively. The detailed derivation was described in Table S6 and **Supporting information S1** and the detailed comparisons were given in Tables 1 and 2. The pure epistatic effects under the two metrics are calculated in the same way and the pure additive and dominance effects under the two metrics are calculated in different ways, here 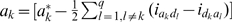 and 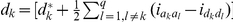.

**Table 2:** Genetic parameter component and parameter estimation method for the genetic models of Z_1_, Z_2_ and Z_3_ under the F_2_ and the F_∞_ metric models.

Data	Model	Model parameter components						Parameter estimation method
		F_2_ metric model			F_∞_ metric model			
		Model mean	Augmented main effect	Augmented epistatic effect	Model mean	Augmented main effect	Augmented epistatic effect	
Z_1_	(3)	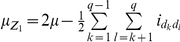	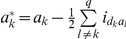	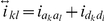	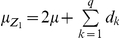	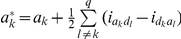	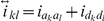	Empirical Bayes
Z_2_	(6)	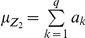	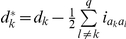	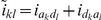	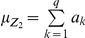	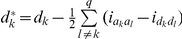	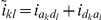	Empirical Bayes
Z_3_	(7)	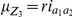	—	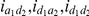	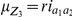	—	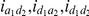	Maximum likelihood

### Genetic parameter estimation

Models (3) and (6) have a uniform appearance. However, the true number of QTL (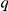) is hard to determine. Variable selection via a stepwise regression or a stochastic search variable selection is the common procedure for epistatic QTL analysis. But these methods are computationally intensive and may not be optimal [31]–[33]. Thus, we adopted the empirical Bayes (E-Bayes) method of Xu [33] for the estimation of parameters in the above models. The E-Bayes approach assumes that there is one QTL standing on each marker throughout the genome and shrinks the genetic effects of all “nonsignificant” QTL toward zero. Here, we only gave some necessary procedures; for the technical details of the E-Bayes refer to the original study of Xu [33].

Models (3) and (6) can be uniformly written as:
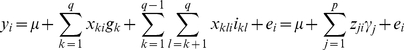
(8)where 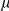 is the model mean; 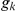 is the augmented main effect of the *k*th QTL; 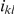 is the augmented epistatic effect between the *k*th and *l*th QTL; 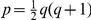 is the total number of genetic effects, including the augmented main and epistatic effects; and 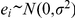 is the residual error. Model (8) can be expressed in matrix form:
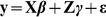
(9)where 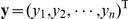; 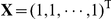; 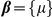; 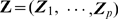; 
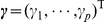
 and 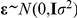.

In the expectation and maximization (EM) algorithm of the E-Bayes method [33], model (9) is a typical mixed model and 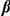 is treated as a fixed effect, whereas 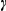 is treated as a random effect. Therefore, 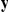 has a multivariate normal distribution with the mean 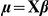 and the variance-covariance matrix 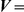 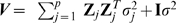.

In the EM algorithm of E-Bayes, the genetic parameters 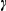 are the focus of interest and the normal prior is assigned to 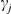, i.e., 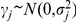 and 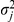 is further assigned a scaled inverse 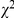 prior, i.e., 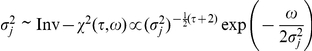. The 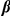 has uniform prior distribution.

The EM algorithm procedures are as follows:

1) Choose 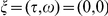 and assign initial values: 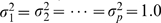, 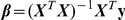, 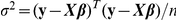.

2) E-step: the best linear unbiased prediction (BLUP) estimation of the expectation of the quadratic term
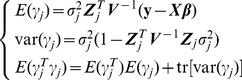
(10)


3) M-step: the maximum-likelihood estimation for 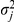, fixed effects and residual variance
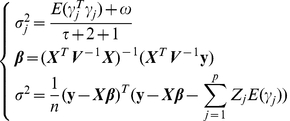
(11)


4) repeat steps 2) - 3) until a certain criterion of convergence is satisfied, e.g. the difference of parameter estimate values between two adjacent iterations were less than 10^−10^.

In addition, we performed a two-dimension scan using the maximum likelihood approach for the estimation parameters in models (7).

### Likelihood ratio test

If we only want to report QTL with relatively large effects and give readers accurate information about how significant the identified QTL were, statistical test should be conducted. The usual likelihood ratio test (LRT) cannot be carried out with the E-Bayes method owing to an oversaturated epistatic genetic model. We proposed the following two-stage selection process to screen the QTL [31]. In the first stage, all QTL with 
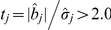
 are picked up. In the second stage, the epistatic genetic model is modified so that only effects past the first round of selection are included in the model. Owing to the smaller dimensionality of the reduced model, we can use the maximum likelihood method to re-analyze the data and perform the LRT [31]. The test statistic is 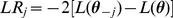
(12)where 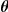 is the parameters vector in the statistical genetic model in the second stage analysis of model (8); 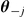 is the parameters vector in 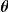 excluding the currently tested genetic effect 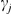; 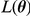 and 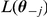 are the log maximum likelihood function for 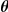 and 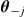, respectively. For simplicity, we took 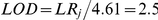 and 3.0 as the critical values in our small and larger genome simulation experiments, respectively.

## Results

### Experiment I

The purpose of the simulation experiment was: (1) to evaluate the statistical performance of the proposed approach; (2) to compare the proposed method with previous approaches, such as Kearsey et al. [12], Frascaroli et al. [16] and Li et al. [17] or Melchinger et al. [7], [21] and Kusterer et al. [22], according to statistical power, standard deviation and accuracy measure; and (3) to compare the TTC design with the F_2_ and F_2:3_ genetic designs.

The simulated genome consisted of three chromosomes (chr1, chr2 and chr3), and 11 evenly spaced markers covered each chromosome with an average marker interval of 10.0 cM. We simulated three main-effect QTL and one pair-wise interaction QTL, all of which overlapped with markers. All three main-effect QTL were located at the center (50.0 cM) of each chromosome, and QTL_2_ on chr2 interacted with QTL_3_ on chr3. The genetic parameters under both the F_2_ and the F_∞_ metric models were as follows: 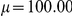; 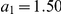 and 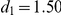 for QTL_1_; 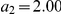 and 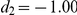 for QTL_2_; 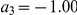 and 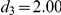 for QTL_3_; 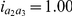, 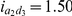, 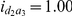 and 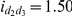 for the epistatic effects between QTL_2_ and QTL_3_. The marginal heritabilities of these genetic effects varied from 1.01% to 36.54%. The sample size (*n*), the number of individual in the F_2_ population, was set at two levels: 200 and 400. The number of individuals (*m*) for each TTC family was set at 1, 5 and 10. The environmental variance (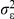) was set at 4.00 and 1.00. To implement the last objective of the simulation experiment, two other kinds of populations, the F_2_ and F_2:3_ populations, were also simulated. However, molecular marker information for all three populations was derived from the corresponding F_2_ individuals. Each treatment was replicated 200 times for the TTC and F_2:3_ designs and 400 times for the F_2_ design. In the analyses of the TTC family data, two approaches were adopted: 1) *Method A*, the proposed method in this study, and 2) *Method B*, the modified method of Kearsey et al. [12], Frascaroli et al. [16] and Li et al. [17] or Melchinger et al. [7], [21] and Kusterer et al. [22], by removing the augmented epistatic effects from models (3) and (6). In the analyses of the F_2_ and F_2:3_ datasets, all of the main effects and all of the pair-wise interaction effects for all of the markers on the whole genome were simultaneously included in the genetic model. For each simulated QTL, we counted the samples in which the LOD statistic was greater than 2.5 and the identified QTL was within 20.0 cM of the simulated QTL. The estimate for QTL parameter was the average of the corresponding estimates in the counted samples. The ratio of the number of such samples to the total number of replicates represented the empirical power of this QTL.

To achieve the first objective of the simulation experiment, 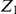, 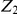 and 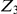 were analyzed by *Method A*. In the first step, with 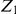 or 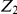 33 augmented additive or dominance effects (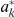 or 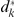) and 528 augmented epistatic effects (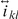 or 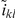) were estimated, and with 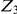 1584 pure epistatic effects (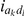,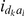 and 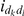) were estimated. All the effects were tested by likelihood ratio statistic in order that real QTL could be identified. The results for detected QTL under the F_2_ metric model were listed in Table 3, Table 4, Table 5. The results show that the newly defined parameters, i.e., 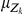, 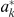, 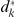 (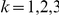), 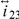 and 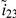, were estimated in an almost unambiguous and unbiased manner, and all of the main-effect QTL were identified with a high statistical power and precision in the estimated effects and positions of the QTL by taking the TTC family mean as the unit of phenotypic measurement. The augmented epistatic QTL (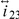 and 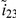) were also well detected, except for the situation when 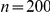, 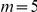 and 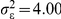. In the second step, all the pure main and epistatic effects would be estimated in an unbiased manner (Table 6). It should also be noted that a large sample (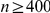), a greater family replication number (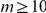), and moderate QTL heritability (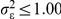) are needed for the partition of the augmented epistatic effects (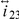 and 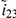) into its components (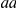, 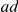, 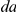 and 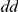), and detecting 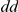 epistasis is more difficult than detecting 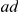 epistasis (Tables 5 and 6). The theoretical explanation is that 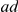 (also 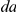) has a larger contribution to the genetic variance of 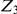 than 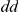 (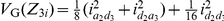 when 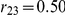, **Supporting Information S2**). In addition, the powers in the detection of the augmented epistatic effects (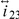 in Table 3 and 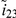 in Table 4) were always much higher than those of pure epistatic effects (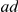, 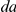 and 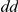 in Table 5). The possible explanations lie in that 1) the augmented epistatic effects (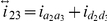 and 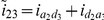) were the sum of two epistatic effects with the same signs in Experiment I and were inflated, and 2) these epistatic effects have different contributions to the genetic variances of 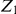, 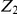 and 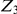 (**Supporting Information S2**).

**Table 3:** Comparison of the proposed approach (Method A) with previous method (Method B) that does not consider augmented epistasis for mapping QTL of *Z*_1_ under the F_2_ metric model.

			Method A**															Method B										
*n*	*m*	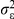	MSe	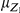	QTL_1_			QTL_2_			QTL_3_			QTL_2_×QTL_3_				MSe	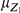	QTL_1_			QTL_2_			QTL_3_		
					*a_1_* *	Position	Power	*a_2_* *	Position	Power	*a_3_* *	Position	Power	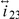	Position_2_	Position_3_	Power			*a_1_*	Position	Power	*a_2_*	Position	Power	*a_3_*	Position	Power
Parameter values				199.25	1.50	50.00		1.50	50.00		-1.75	50.00		2.50	50.00	50.00				1.50	50.00		2.00	50.00		-1.00	50.00	
200	1	4.00	13.321(1.370)	200.438(0.514)	1.641(0.246)	50.135(8.410)	0.740	1.625(0.290)	49.937(7.779)	0.790	-1.808(0.312)	50.160(7.069)	0.935	4.218(0.539)	40.000(8.165)	52.500(12.583)	0.020	13.363(1.357)	200.502(0.258)	1.648(0.251)	50.068(7.781)	0.740	1.631(0.286)	50.127(7.249)	0.785	-1.827(0.304)	50.437(7.018)	0.915
		1.00	7.013(0.727)	200.387(0.477)	1.508(0.241)	49.682(4.104)	0.980	1.511(0.249)	50.459(4.332)	0.980	-1.779(0.258)	50.103(3.311)	1.000	3.143(0.400)	47.692(8.321)	51.538(6.887)	0.065	7.053(0.695)	200.503(0.199)	1.510(0.240)	49.887(3.857)	0.980	1.511(0.252)	50.606(4.899)	0.990	-1.779(0.259)	50.047(3.670)	1.000
	5	4.00	2.643(0.335)	199.638(0.681)	1.485(0.161)	50.061(0.725)	0.990	1.505(0.166)	49.950(0.707)	1.000	-1.733(0.154)	50.048(0.478)	1.000	2.689(0.378)	50.112(3.482)	49.346(4.197)	0.630	2.933(0.305)	200.495(0.118)	1.500(0.163)	50.010(1.017)	0.990	1.517(0.182)	50.027(0.382)	0.995	-1.736(0.170)	50.045(0.454)	1.000
		1.00	1.351(0.145)	199.261(0.151)	1.498(0.111)	50.011(0.156)	1.000	1.479(0.126)	49.991(0.573)	1.000	-1.744(0.109)	49.985(0.217)	1.000	2.499(0.243)	50.201(2.000)	49.749(1.863)	0.995	1.732(0.165)	200.509(0.100)	1.489(0.135)	49.932(0.752)	1.000	1.485(0.151)	49.994(0.546)	1.000	-1.738(0.130)	49.986(0.282)	1.000
	10	4.00	1.270(0.138)	199.268(0.219)	1.505(0.118)	50.000(0.000)	1.000	1.492(0.116)	50.000(0.000)	1.000	-1.746(0.097)	49.998(0.235)	1.000	2.494(0.279)	49.795(2.479)	50.051(1.899)	0.975	1.651(0.159)	200.506(0.090)	1.504(0.133)	49.984(0.353)	1.000	1.487(0.159)	50.000(0.000)	1.000	-1.750(0.129)	49.984(0.350)	1.000
		1.00	0.679(0.076)	199.247(0.115)	1.496(0.078)	50.007(0.099)	1.000	1.494(0.082)	50.011(0.160)	1.000	-1.752(0.073)	50.018(0.195)	1.000	2.515(0.170)	50.000(0.000)	49.934(0.740)	0.990	1.067(0.098)	200.506(0.073)	1.502(0.104)	50.011(0.438)	1.000	1.502(0.132)	50.008(0.112)	1.000	-1.742(0.114)	50.021(0.299)	1.000
400	1	4.00	13.101(1.008)	200.250(0.639)	1.522(0.233)	50.202(4.828)	0.990	1.534(0.242)	49.462(2.959)	0.995	-1.755(0.246)	50.000(3.023)	0.990	3.241(0.333)	50.000(5.872)	49.667(4.901)	0.150	13.207(1.003)	200.488(0.187)	1.522(0.236)	49.898(3.911)	0.985	1.530(0.243)	49.463(2.959)	1.000	-1.760(0.245)	49.950(2.930)	0.995
		1.00	6.797(0.485)	199.647(0.667)	1.502(0.176)	50.000(1.743)	0.990	1.475(0.181)	50.017(1.439)	1.000	-1.726(0.185)	50.098(1.001)	0.995	2.650(0.302)	50.156(5.468)	49.688(4.514)	0.640	7.082(0.442)	200.504(0.131)	1.501(0.184)	50.101(1.740)	0.990	1.479(0.180)	50.108(1.369)	0.990	-1.733(0.194)	50.078(0.776)	0.990
	5	4.00	2.544(0.185)	199.255(0.210)	1.515(0.125)	50.014(0.192)	1.000	1.498(0.098)	49.997(0.301)	1.000	-1.745(0.120)	49.989(0.330)	1.000	2.483(0.283)	50.136(2.150)	50.035(1.612)	0.985	2.924(0.209)	200.500(0.082)	1.517(0.130)	50.020(0.281)	1.000	1.498(0.110)	50.029(0.526)	1.000	-1.746(0.136)	49.996(0.405)	1.000
		1.00	1.349(0.102)	199.246(0.110)	1.499(0.076)	50.007(0.242)	1.000	1.509(0.068)	50.000(0.000)	1.000	-1.761(0.073)	50.000(0.000)	1.000	2.495(0.162)	49.987(0.180)	49.994(0.090)	1.000	1.733(0.106)	200.503(0.063)	1.502(0.090)	50.003(0.284)	1.000	1.509(0.091)	50.014(0.202)	1.000	-1.757(0.095)	50.000(0.000)	1.000
	10	4.00	1.281(0.087)	199.240(0.139)	1.504(0.077)	50.000(0.000)	1.000	1.503(0.078)	49.993(0.168)	1.000	-1.750(0.078)	50.012(0.123)	1.000	2.506(0.204)	50.000(0.000)	49.938(0.617)	1.000	1.674(0.111)	200.498(0.065)	1.501(0.090)	50.000(0.000)	1.000	1.494(0.098)	50.000(0.000)	1.000	-1.749(0.104)	50.013(0.127)	1.000
		1.00	0.677(0.055)	199.250(0.081)	1.501(0.052)	50.000(0.000)	1.000	1.493(0.054)	50.000(0.000)	1.000	-1.754(0.053)	49.994(0.079)	1.000	2.505(0.118)	49.991(0.126)	50.009(0.126)	1.000	1.064(0.067)	200.505(0.053)	1.500(0.068)	50.000(0.000)	1.000	1.495(0.082)	50.004(0.062)	1.000	-1.758(0.088)	49.994(0.089)	1.000

* *n* denotes sample size; *m* is number of replications; and 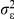 is residual variance for the phenotypic trait value 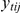. The numbers in parentheses are standard deviation and the same is true for the later tables except for Table 6 to Table 8.

** 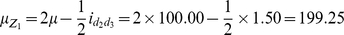, 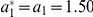, 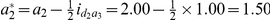, 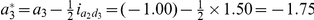 and 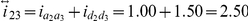, see model (3) for details.

**Table 4:** Comparison of the proposed approach (Method A) with previous method (Method B) that does not consider augmented epistasis for mapping QTL of *Z*_2_ under the F_2_ metric model.

			Method A**															Method B										
*n*	*m*	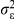	MSe	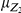	QTL_1_			QTL_2_			QTL_3_			QTL_2_×QTL_3_				MSe	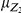	QTL_1_			QTL_2_			QTL_3_		
					*d*_1_ *	Position	Power	*d*_2_ *	Position	Power	*d*_3_ *	Position	Power	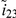	Position_2_	Position_3_	Power			*d*_1_	Position	Power	*d*_2_	Position	Power	*d*_3_	Position	Power
Parameter values				2.50	1.50	50.00		-1.50	50.00		1.50	50.00		2.50	50.00	50.00				1.50	50.00		-1.00	50.00		2.00	50.00	
200	1	4.00	13.033(1.479)	2.566(0.351)	1.658(0.275)	49.351(6.635)	0.770	-1.631(0.275)	49.379(8.184)	0.725	1.617(0.284)	49.737(8.608)	0.760	4.019(0.506)	51.429(10.885)	45.143(14.219)	0.175	13.297(1.424)	2.525(0.252)	1.661(0.292)	49.545(5.906)	0.785	-1.636(0.282)	49.801(7.346)	0.755	1.632(0.292)	49.400(7.877)	0.750
		1.00	6.834(0.761)	2.513(0.227)	1.518(0.237)	50.127(4.821)	0.960	-1.511(0.240)	49.848(4.342)	0.985	1.518(0.235)	50.084(5.822)	0.990	3.002(0.447)	53.133(11.469)	49.157(10.146)	0.415	7.116(0.741)	2.509(0.192)	1.516(0.237)	50.052(4.007)	0.970	-1.520(0.237)	49.846(4.245)	0.975	1.529(0.223)	49.949(5.392)	0.985
	5	4.00	2.525(0.276)	2.485(0.117)	1.516(0.155)	50.003(0.836)	1.000	-1.496(0.166)	49.942(1.187)	1.000	1.494(0.166)	49.899(1.000)	0.995	2.537(0.416)	50.011(5.056)	50.320(5.746)	0.905	2.906(0.299)	2.479(0.121)	1.527(0.157)	49.982(1.385)	1.000	-1.495(0.184)	49.899(0.926)	0.995	1.512(0.177)	49.997(0.036)	0.995
		1.00	1.338(0.146)	2.502(0.096)	1.486(0.114)	49.926(0.533)	1.000	-1.490(0.098)	49.978(0.313)	1.000	1.507(0.108)	50.033(0.308)	1.000	2.489(0.260)	49.571(2.067)	50.236(2.416)	1.000	1.721(0.163)	2.503(0.087)	1.482(0.137)	49.926(0.956)	0.995	-1.488(0.130)	50.031(0.661)	0.995	1.515(0.135)	50.044(0.629)	0.995
	10	4.00	1.269(0.132)	2.512(0.109)	1.497(0.104)	50.022(0.306)	1.000	-1.507(0.126)	50.004(0.311)	1.000	1.497(0.109)	50.010(0.451)	1.000	2.530(0.319)	50.045(2.162)	50.239(2.571)	1.000	1.673(0.150)	2.510(0.093)	1.498(0.122)	50.018(0.261)	1.000	-1.518(0.147)	50.021(0.299)	1.000	1.495(0.128)	50.028(0.490)	0.995
		1.00	0.686(0.075)	2.502(0.070)	1.496(0.079)	50.000(0.000)	1.000	-1.498(0.075)	49.993(0.098)	1.000	1.506(0.073)	49.994(0.092)	1.000	2.490(0.186)	49.967(0.471)	50.017(0.238)	0.995	1.073(0.096)	2.501(0.071)	1.502(0.096)	50.000(0.000)	1.000	-1.495(0.122)	50.041(0.480)	1.000	1.515(0.118)	50.000(0.000)	1.000
400	1	4.00	12.764(1.022)	2.473(0.245)	1.523(0.238)	50.282(3.733)	0.990	-1.487(0.237)	50.063(4.238)	0.995	1.504(0.239)	49.594(4.020)	0.985	2.995(0.478)	50.938(8.835)	49.792(9.059)	0.480	13.128(0.980)	2.486(0.186)	1.515(0.251)	50.355(3.867)	0.995	-1.503(0.225)	49.899(4.505)	0.990	1.519(0.239)	49.848(4.331)	0.990
		1.00	6.807(0.523)	2.510(0.143)	1.498(0.174)	50.127(1.460)	0.990	-1.475(0.185)	50.394(2.379)	0.995	1.478(0.171)	49.952(1.959)	1.000	2.574(0.376)	49.586(6.110)	50.296(6.585)	0.845	7.179(0.498)	2.506(0.137)	1.497(0.170)	50.115(1.763)	0.995	-1.471(0.188)	50.151(1.582)	0.995	1.480(0.183)	49.789(1.730)	1.000
	5	4.00	2.544(0.195)	2.510(0.097)	1.498(0.116)	49.981(0.203)	1.000	-1.499(0.111)	50.000(0.000)	1.000	1.494(0.124)	50.020(0.390)	1.000	2.472(0.309)	49.897(2.057)	50.398(3.529)	0.990	2.933(0.212)	2.508(0.080)	1.495(0.133)	49.984(0.163)	1.000	-1.500(0.131)	49.994(0.301)	1.000	1.490(0.143)	50.000(0.000)	1.000
		1.00	1.348(0.101)	2.500(0.075)	1.502(0.081)	49.991(0.262)	1.000	-1.511(0.076)	50.009(0.131)	1.000	1.497(0.077)	50.016(0.165)	1.000	2.507(0.178)	50.017(0.235)	49.983(0.235)	1.000	1.736(0.110)	2.500(0.065)	1.502(0.100)	49.985(0.216)	1.000	-1.510(0.103)	49.991(0.130)	1.000	1.497(0.096)	50.000(0.000)	1.000
	10	4.00	1.261(0.089)	2.504(0.071)	1.500(0.079)	50.029(0.240)	1.000	-1.503(0.076)	49.996(0.058)	1.000	1.499(0.074)	50.000(0.000)	1.000	2.489(0.208)	50.022(0.249)	49.967(0.326)	1.000	1.650(0.117)	2.506(0.071)	1.498(0.093)	50.028(0.236)	1.000	-1.510(0.099)	50.000(0.258)	1.000	1.501(0.098)	50.002(0.215)	1.000
		1.00	0.677(0.054)	2.496(0.065)	1.504(0.058)	50.007(0.092)	1.000	-1.491(0.056)	50.000(0.000)	1.000	1.502(0.054)	49.993(0.068)	1.000	2.512(0.128)	50.012(0.163)	49.994(0.082)	1.000	1.069(0.074)	2.498(0.051)	1.502(0.068)	49.998(0.167)	1.000	-1.490(0.083)	49.996(0.166)	1.000	1.500(0.087)	49.996(0.054)	1.000

* *n* denotes sample size; *m* is number of replications; and 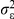 is residual variance for the phenotypic trait value 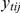.

** 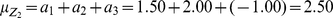, 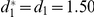, 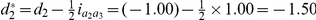, 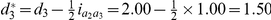 and 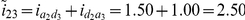, see Model (6) for details.

**Table 5:** Mapping QTL for *Z*_3_ under the F_2_ metric model.

*n*	*m*	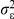	MSe	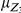	QTL_2_×QTL_3_							
					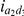	Power	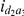	Power	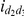	Power	Position_2_	Position_3_
Parameter values				0.50	1.50		1.00		1.50		50.00	50.00
200	1	4.00	49.410(4.534)	0.508(0.515)	4.283(0.377)	0.045	5.353(0.376)	0.010	6.659(1.376)	0.025	50.250(8.293)	49.450(7.843)
		1.00	32.103(3.311)	0.535(0.396)	3.716(0.235)	0.060	4.208(0.840)	0.015	4.751(0.281)	0.010	50.050(8.175)	50.600(7.274)
	5	4.00	9.993(0.981)	0.498(0.218)	2.499(0.254)	0.155	2.302(0.300)	0.030	3.471(0.421)	0.045	49.750(7.120)	50.050(6.458)
		1.00	6.367(0.609)	0.514(0.175)	2.054(0.244)	0.320	1.932(0.233)	0.120	2.698(0.324)	0.110	49.900(6.260)	50.350(5.050)
	10	4.00	4.961(0.502)	0.509(0.158)	1.809(0.253)	0.440	1.748(0.222)	0.135	2.336(0.300)	0.150	49.950(5.888)	49.850(4.424)
		1.00	3.158(0.338)	0.505(0.120)	1.627(0.252)	0.815	1.392(0.178)	0.310	2.088(0.306)	0.370	49.650(4.179)	50.150(3.396)
400	1	4.00	50.246(3.427)	0.489(0.350)	3.511(0.393)	0.080	3.556(0.184)	0.020	5.020(0.519)	0.050	49.800(7.432)	50.100(7.434)
		1.00	31.734(2.121)	0.511(0.271)	2.838(0.406)	0.150	2.778(0.332)	0.045	4.008(0.685)	0.040	50.250(7.328)	49.550(6.821)
	5	4.00	10.052(0.675)	0.500(0.152)	1.903(0.253)	0.460	1.739(0.182)	0.135	2.534(0.267)	0.165	50.900(5.947)	50.250(4.853)
		1.00	6.391(0.489)	0.515(0.123)	1.627(0.260)	0.800	1.450(0.191)	0.225	2.009(0.289)	0.350	49.850(4.646)	50.300(3.739)
	10	4.00	5.003(0.386)	0.506(0.124)	1.540(0.277)	0.915	1.319(0.190)	0.375	1.882(0.256)	0.490	50.100(3.750)	50.300(2.820)
		1.00	3.174(0.222)	0.495(0.081)	1.495(0.246)	0.995	1.117(0.179)	0.755	1.633(0.263)	0.820	50.400(2.981)	50.250(1.859)

* *n* denotes sample size; *m* is family replication number; and 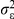 is residual variance for the phenotypic trait value 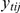.

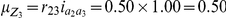
, see Model (7) for details.

**Table 6:** Estimation of pure main and epistatic effects of QTL in the F_2_-based TTC design using the two-step approach under the cases of *n* = 400, *m* = 10 and 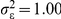 (200 replicates).

Metric	Statistics	QTL_1_		QTL_2_		QTL_3_		QTL_2_×QTL_3_			
		*a*_1_	*d*_1_	*a*_2_	*d*_2_	*a*_3_	*d*_3_	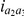	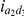	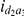	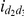
Parameter values		1.50	1.50	2.00	-1.00	-1.00	2.00	1.00	1.50	1.00	1.50
F_2_	Mean	1.501	1.504	2.028	-1.128	-1.025	1.865	0.886	1.466	1.075	1.633
	SD	0.052	0.058	0.108	0.214	0.100	0.214	0.262	0.200	0.190	0.263
	Power	1.000	1.000	1.000	1.000	1.000	1.000	0.820	0.995	0.995	0.820
F_∞_	Mean	1.502	1.504	2.049	-1.051	-1.062	1.940	0.797	1.468	1.080	1.724
	SD	0.055	0.063	0.213	0.305	0.193	0.306	0.263	0.224	0.219	0.264
	Power	1.000	1.000	1.000	1.000	1.000	1.000	0.670	0.990	0.990	0.670

To achieve the second objective of the simulation experiment, 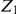 and 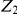 were re-analyzed by *method B* and the results under the F_2_ metric model were also listed in Tables 3 and 4. The results show that the *Z*_1_ and *Z*_2_ could still be used to unbiasedly estimate QTL additive (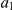) and dominance effect (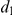) when the QTL (QTL_1_) acted independently; but provided biased estimation of QTL additive (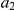 and 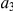) and dominance effects (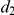 and 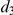) when the QTL acted dependently (QTL_2_ and QTL_3_). The additive (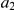 and 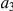) and dominance effects (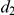 and 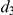) of interactive QTL obtained by *Method B* in Tables 3 and 4 were indeed the newly defined additive effects (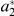 and 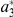) and the new dominance effects (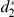 and 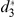) with slightly poorer precision (little larger in standard deviation) in estimated QTL effects and positions and lower statistical power. This means that the new method was better than the previous methods of Kearsey et al. [12], Frascaroli et al. [16] and Li et al. [17] in the presence of epistasis. The higher statistical power and smaller error variance for *method A* over *method B* shows that the new method was also superior to the methods of Melchinger et al. [7], [21] and Kusterer et al. [22].

To achieve the third objective of the simulation experiment, the F_2_ and F_2:3_ data were analyzed and the results under the F_2_ metric model were listed in Tables 7 and 8. The results show that many effects could be estimated in an unambiguous and unbiased manner in the F_2_ and F_2:3_ genetic designs. In the situation of 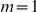, the F_2_ design was superior to the both TTC and F_2:3_ designs. The reasons are as follows. In all the above three designs, marker genotypes were from F_2_ individuals. If 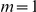, genotype sampling error was large for both TTC and F_2:3_ designs. Meanwhile, the proposed approach in this study did not consider the mixed distribution of the F_2:3_ (or TTC) progeny derived from heterozygous F_2_ parents. However, the powers in the detection of the main and epistatic QTL were smaller for the F_2_ design than for the TTC design with 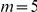 (or 10) when sample size (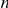) was small and/or environmental variance (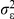) was large, and the same trend was obtained for the precision of the estimates for the effects and the positions of the main and epistatic QTL. For example, when 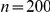 and 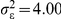, the power for main effects 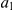 and 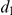 were 0.850 and 0.775 and the standard deviation (SD) were 0.253 and 0.308, respectively, in F_2_ design (Table 7); while the power for 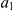 and 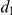 were 1.000 and 1.000 and the SD were 0.118 and 0.104, respectively, in TTC design with a family replication of 10 (Tables 3 and 4). This may be due to the fact that the phenotypic value is measured from F_2_ individuals and from the TTC family, and the family mean can be used to decrease the residual variance and to improve the precision of the phenotypic data. Both the TTC and F_2:3_ designs use family mean to decrease environmental variance and improve the precision of phenotype of quantitative trait. In addition, the dominant components decrease significantly in the F_2:3_ design due to its self-crossing, and the statistical powers for detecting dominance effects, additive by dominance (dominance by additive) epistatic effect and especially dominance by dominance epistatic effect in the F_2:3_ design will be lower than that in the TTC design. For example, when 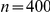, 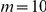 and 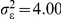, the power of 0.170 for 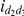 in F_2:3_ (Table 8) was much lower than that of 0.490 in the TTC (Table 5). The genetic variance contributed by the simulated three QTL under TTC and F_2:3_ designs were (**Supporting Information S2**):
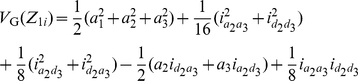


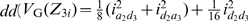


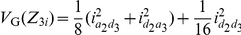


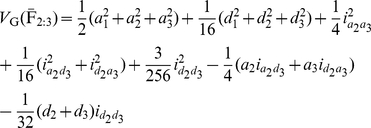



**Table 7:** Results of QTL mapping in F_2_ population under the F_2_ metric model (400 replications).

*n*	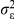	Statistics	MSe	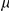	QTL_1_			QTL_2_			QTL_3_			QTL_2_×QTL_3_					
					*a_1_*	*d_1_*	Position	*a_2_*	*d_2_*	Position	*a_3_*	*d_3_*	Position	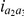	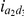	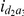	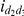	Position_2_	Position_3_
Parameter values				100.00	1.50	1.50	50.00	2.00	-1.00	50.00	-1.00	2.00	50.00	1.00	1.50	1.00	1.50	50.00	50.00
200	4.00	Mean	4.016	100.051	1.480	1.571	50.193	1.920	-1.267	49.951	-1.036	1.940	50.138	1.320	1.813	1.637	2.317	50.413	50.233
		SD	0.613	0.336	0.253	0.308	2.522	0.307	0.220	2.059	0.197	0.357	3.825	0.225	0.335	0.255	0.370	9.245	8.089
		Power			0.850	0.775		0.963	0.313		0.488	0.935		0.418	0.540	0.158	0.200		
	1.00	Mean	0.979	99.984	1.469	1.479	50.013	1.979	-0.971	50.077	-0.961	1.967	49.962	0.980	1.485	1.032	1.516	50.086	50.058
		SD	0.137	0.142	0.133	0.179	0.271	0.132	0.160	0.867	0.141	0.184	1.649	0.178	0.272	0.193	0.311	2.974	2.940
		Power			0.998	0.993		1.000	0.920		0.923	0.998		0.960	0.995	0.730	0.848		
400	4.00	Mean	3.952	99.963	1.465	1.495	49.922	1.974	-1.039	49.920	-0.984	2.001	49.894	1.058	1.548	1.258	1.768	50.180	50.311
		SD	0.340	0.207	0.202	0.211	1.130	0.191	0.184	1.757	0.156	0.231	2.018	0.226	0.313	0.238	0.304	5.795	4.453
		Power			0.973	0.963		1.000	0.740		0.808	1.000		0.783	0.893	0.425	0.525		
	1.00	Mean	0.970	99.995	1.498	1.504	49.998	1.997	-0.987	49.999	-0.994	2.000	50.005	0.995	1.502	0.997	1.531	49.959	49.952
		SD	0.079	0.065	0.078	0.111	0.080	0.085	0.111	0.090	0.089	0.111	0.369	0.119	0.166	0.163	0.237	0.966	1.364
		Power			1.000	1.000		1.000	0.993		0.998	1.000		0.995	1.000	0.985	0.998		

* *n* denotes sample size; and 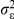 is residual variance for the phenotypic trait value 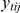.

**Table 8:** Results of QTL mapping in F_2:3_ population under the F_2_ metric model (200 replications)

*n*	*m*	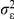	Statistics	MSe	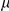	QTL_1_			QTL_2_			QTL_3_			QTL_2_×QTL_3_					
						*a*_1_	*d*_1_	Position	*a*_2_	*d*_2_	Position	*a*_3_	*d*_3_	Position	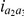	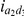	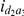	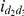	Position_2_	Position_3_
Parameter values					100.00	1.50	1.50	50.00	2.00	-1.00	50.00	-1.00	2.00	50.00	1.00	1.50	1.00	1.50	50.00	50.00
200	1	4.00	Mean	7.447	99.577	1.555	3.266	49.298	1.644	-2.965	50.408	-1.432	3.056	48.289	1.568	3.661	3.724	6.774	49.180	49.727
			SD	0.992	0.442	0.350	0.363	6.508	0.303	0.182	5.546	0.256	0.396	9.174	0.279	.	0.756	0.673	15.060	11.073
			Power			0.260	0.035		0.485	0.015		0.160	0.040		0.130	0.005	0.190	0.010		
		1.00	Mean	4.598	99.659	1.520	2.492	49.739	1.629	-2.321	50.556	-1.316	2.495	49.191	1.213	2.676	3.005	5.471	48.164	50.340
			SD	0.625	0.417	0.276	0.431	5.123	0.303	0.251	4.390	0.218	0.383	5.834	0.191	0.247	0.570	0.689	16.695	10.972
			Power			0.535	0.095		0.720	0.015		0.260	0.095		0.320	0.015	0.260	0.015		
	5	4.00	Mean	1.647	99.756	1.451	1.875	49.996	1.688	-1.675	50.053	-1.205	1.874	49.470	0.986	2.023	2.139	3.924	50.490	49.984
			SD	0.228	0.327	0.205	0.450	1.910	0.284	0.303	3.002	0.216	0.352	4.288	0.171	0.413	0.520	1.001	7.294	7.627
			Power			0.810	0.150		0.950	0.165		0.630	0.350		0.785	0.155	0.265	0.030		
		1.00	Mean	0.958	99.825	1.449	1.601	49.905	1.780	-1.464	49.805	-1.132	1.743	49.969	0.973	1.666	1.684	3.168	49.669	50.851
			SD	0.156	0.344	0.170	0.314	1.741	0.258	0.302	2.049	0.222	0.387	3.680	0.142	0.307	0.409	0.453	4.729	5.057
			Power			0.965	0.405		0.965	0.375		0.780	0.570		0.975	0.415	0.255	0.105		
	10	4.00	Mean	0.798	99.912	1.486	1.562	50.019	1.808	-1.411	50.094	-1.119	1.661	50.083	0.970	1.602	1.542	2.957	49.405	49.872
			SD	0.122	0.282	0.113	0.259	0.946	0.245	0.241	1.606	0.188	0.316	2.209	0.157	0.305	0.443	0.601	4.014	5.857
			Power			0.980	0.585		0.990	0.370		0.795	0.700		0.975	0.510	0.290	0.110		
		1.00	Mean	0.480	99.878	1.485	1.524	50.077	1.895	-1.369	50.004	-1.102	1.598	50.090	0.971	1.462	1.163	2.738	50.324	50.007
			SD	0.087	0.270	0.103	0.249	0.861	0.200	0.229	1.200	0.190	0.332	1.135	0.099	0.294	0.287	0.661	2.936	3.537
			Power			0.975	0.710		1.000	0.650		0.935	0.835		1.000	0.800	0.395	0.120		
400	1	4.00	Mean	7.535	99.635	1.449	2.241	49.375	1.668	-2.508	49.907	-1.263	2.500	49.643	1.145	2.979	2.809	5.061	47.713	49.927
			SD	0.621	0.365	0.233	0.304	3.559	0.315	0.580	3.263	0.218	0.383	4.957	0.187	0.316	0.532	0.533	10.857	9.961
			Power			0.535	0.055		0.730	0.050		0.330	0.110		0.510	0.050	0.300	0.015		
		1.00	Mean	4.405	99.759	1.490	1.940	50.294	1.639	-1.831	49.843	-1.195	2.013	50.567	1.017	2.514	2.231	4.379	50.307	50.276
			SD	0.382	0.368	0.208	0.351	3.063	0.287	0.269	1.892	0.313	0.392	5.326	0.169	0.375	0.545	0.364	9.151	6.021
			Power			0.785	0.225		0.900	0.125		0.545	0.295		0.860	0.100	0.250	0.020		
	5	4.00	Mean	1.486	99.888	1.465	1.543	50.217	1.848	-1.448	49.858	-1.130	1.764	50.073	0.994	1.555	1.502	3.301	50.372	49.942
			SD	0.142	0.271	0.142	0.277	1.682	0.242	0.267	1.327	0.210	0.423	1.339	0.137	0.300	0.375	0.895	3.409	4.417
			Power			0.935	0.640		0.990	0.485		0.850	0.730		1.000	0.615	0.310	0.205		
		1.00	Mean	0.879	99.869	1.486	1.505	50.073	1.933	-1.424	49.963	-1.080	1.639	49.998	0.994	1.452	1.161	2.852	50.372	50.199
			SD	0.089	0.222	0.092	0.247	0.795	0.159	0.224	0.490	0.200	0.344	1.347	0.080	0.289	0.272	0.927	2.272	3.123
			Power			0.985	0.720		1.000	0.740		0.950	0.895		1.000	0.890	0.505	0.090		
	10	4.00	Mean	0.740	99.923	1.499	1.504	50.062	1.941	-1.389	49.950	-1.081	1.665	50.006	0.994	1.437	1.104	2.945	49.811	49.771
			SD	0.065	0.178	0.075	0.228	0.720	0.156	0.208	0.707	0.163	0.351	0.508	0.089	0.283	0.223	0.845	2.789	2.498
			Power			1.000	0.905		1.000	0.740		0.935	0.960		1.000	0.925	0.625	0.170		
		1.00	Mean	0.429	99.936	1.497	1.487	49.966	1.982	-1.355	49.983	-1.029	1.715	50.008	0.998	1.473	0.955	2.409	49.862	49.872
			SD	0.036	0.139	0.060	0.215	0.626	0.109	0.178	0.185	0.091	0.284	0.080	0.050	0.221	0.181	0.736	1.653	2.063
			Power			1.000	0.965		1.000	0.865		1.000	0.990		1.000	0.995	0.930	0.180		

* *n* denotes sample size; *m* is family replication number; and 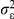 is residual variance for the phenotypic trait value 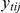.

These variance component can be used to interpret the above simulated experiments results.

### Experiment II

The purpose of the simulation experiment was to show the statistical properties of the proposed approach in the TTC design when the augmented epistatic effects consisted of two epistatic effects of equal strength in opposite directions. The genetic parameters under both the F_2_ and the F_∞_ the metric models were as follows: 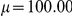; 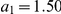, 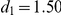 for QTL_1_; 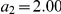, 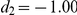 for QTL_2_; 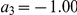, 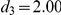 for QTL_3_; 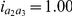, 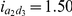, 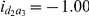 and 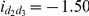 for the epistatic effects between QTL_2_ and QTL_3_. The marginal heritabilities of these genetic effects now varied from 0.98% to 38.75%. The value of *m* was set at 5 and 10. The other settings were the same as those in Experiments I.

The results for Experiments II are listed in Table 9, Table 10, Table 11. The results show that the powers in the detection of the augmented epistatic effects (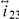 in Table 9 and 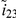 in Table 10) were very low. The results are reasonable because the genetic contributions of the augmented epistatic effects to the genetic variance of 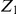 and 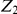 were low. However, the powers for pure epistatic effects (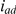, 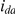 and 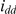) remained steady (Tables 5 and 11) because the genetic contributions for these effects do not change.

**Table 9:** Results of mapping QTL of *Z*_1_ under F_2_ metric model while augmented epistatic effects consisted of two epistatic effects of equal strength in opposite directions (200 replications).

*n*	*m*	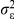	MSe	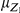	QTL_1_			QTL_2_			QTL_3_			QTL_2_×QTL_3_			
					*a_1_* *	Position	Power	*a_2_* *	Position	Power	*a_3_* *	Position	Power	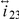	Position_2_	Position_3_	Power
Parameter values				200.75	1.50	50.00		2.50	50.00		-1.75	50.00		-0.50	50.00	50.00	
200	5	4.00	2.567(0.280)	200.502(0.151)	1.488(0.162)	49.977(0.322)	1.000	2.501(0.152)	49.973(0.280)	1.000	-1.740(0.174)	50.000(0.000)	1.000				0.000
		1.00	1.349(0.143)	200.502(0.107)	1.496(0.103)	50.028(0.535)	1.000	2.503(0.096)	50.000(0.000)	1.000	-1.748(0.101)	50.000(0.000)	1.000				0.000
	10	4.00	1.264(0.128)	200.506(0.101)	1.496(0.108)	50.007(0.337)	1.000	2.494(0.109)	49.998(0.144)	1.000	-1.753(0.121)	49.987(0.244)	1.000	-1.079	40.000	40.000	0.005
		1.00	0.684(0.081)	200.514(0.121)	1.513(0.081)	49.969(0.253)	1.000	2.498(0.064)	49.995(0.065)	1.000	-1.749(0.077)	50.019(0.202)	1.000	-0.888(0.096)	53.333(15.275)	43.333(5.774)	0.015
400	5	4.00	2.530(0.193)	200.515(0.127)	1.494(0.113)	50.009(0.576)	1.000	2.504(0.107)	49.994(0.088)	1.000	-1.744(0.126)	50.051(0.362)	1.000				0.000
		1.00	1.337(0.099)	200.507(0.104)	1.500(0.080)	50.000(0.000)	1.000	2.501(0.066)	49.995(0.070)	1.000	-1.751(0.078)	49.985(0.208)	1.000				0.000
	10	4.00	1.275(0.100)	200.517(0.127)	1.488(0.079)	50.000(0.000)	1.000	2.492(0.073)	50.000(0.077)	1.000	-1.750(0.079)	49.999(0.216)	1.000	-0.880(0.036)	60.000(15.492)	50.000(21.909)	0.030
		1.00	0.677(0.057)	200.512(0.088)	1.503(0.056)	49.996(0.057)	1.000	2.500(0.049)	50.000(0.000)	1.000	-1.756(0.059)	50.000(0.000)	1.000	-0.676(0.074)	52.500(14.880)	45.000(5.345)	0.040

* *n* denotes sample size; *m* is family replication number; and 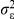 is residual variance for the phenotypic trait value 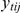.

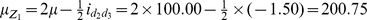
, 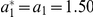, 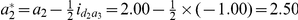, 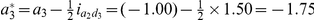 and 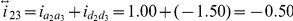, see Model (3) for details.

**Table 10:** Results of mapping QTL of *Z*_2_ under the F_2_ metric model while augmented epistatic effects consisted of two epistatic effects of equal strength in opposite directions (200 replications).

*n*	*m*	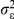	MSe	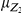	QTL_1_			QTL_2_			QTL_3_			QTL_2_×QTL_3_			
					*d_1_* *	Position	Power	*d_2_* *	Position	Power	*d_3_* *	Position	Power	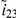	Position_2_	Position_3_	Power
Parameter values				2.50	1.50	50.00		-1.50	50.00		1.50	50.00		0.50	50.00	50.00	
200	5	4.00	2.501(0.266)	2.513(0.153)	1.483(0.175)	50.032(1.635)	1.000	-1.512(0.146)	49.935(1.198)	0.995	1.526(0.156)	50.014(0.920)	0.995	1.605(0.074)	50.000(10.000)	60.000(20.000)	0.015
		1.00	1.327(0.151)	2.495(0.111)	1.496(0.098)	49.981(0.515)	1.000	-1.500(0.109)	50.045(0.375)	1.000	1.482(0.105)	50.007(0.564)	1.000	1.198(0.226)	50.000(14.142)	62.500(17.078)	0.020
	10	4.00	1.271(0.135)	2.500(0.113)	1.501(0.119)	50.028(0.306)	1.000	-1.507(0.113)	49.994(0.235)	1.000	1.491(0.129)	49.979(0.299)	1.000	1.141(0.181)	41.429(22.678)	54.286(9.759)	0.035
		1.00	0.674(0.080)	2.500(0.078)	1.496(0.075)	50.000(0.155)	1.000	-1.498(0.070)	49.990(0.139)	1.000	1.496(0.074)	50.000(0.000)	1.000	0.852(0.127)	51.818(15.374)	44.545(11.282)	0.055
400	5	4.00	2.519(0.199)	2.507(0.120)	1.498(0.123)	50.000(0.000)	1.000	-1.504(0.107)	49.986(0.195)	1.000	1.515(0.113)	50.017(0.242)	1.000	1.218(0.184)	52.500(19.086)	47.500(12.817)	0.040
		1.00	1.327(0.102)	2.498(0.071)	1.502(0.084)	50.008(0.253)	1.000	-1.502(0.065)	50.002(0.165)	1.000	1.514(0.075)	49.990(0.209)	1.000	0.896(0.112)	54.286(7.868)	48.571(12.150)	0.035
	10	4.00	1.277(0.084)	2.509(0.097)	1.500(0.076)	50.029(0.234)	1.000	-1.490(0.072)	49.987(0.233)	1.000	1.501(0.074)	49.991(0.123)	1.000	0.909(0.103)	46.250(11.726)	50.000(10.215)	0.120
		1.00	0.669(0.049)	2.505(0.059)	1.501(0.052)	50.003(0.045)	1.000	-1.500(0.046)	50.000(0.000)	1.000	1.499(0.056)	50.003(0.046)	1.000	0.647(0.060)	50.208(11.938)	48.750(9.368)	0.240

* *n* denotes sample size; *m* is family replicationnumber; and 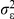 is residual variance for the phenotypic trait value 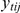.

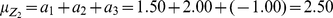
, 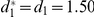, 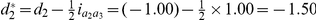, 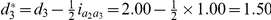 and 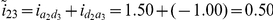, see Model (6) for details.

**Table 11:** Results of mapping QTL of *Z*_3_ under F_2_ metric model while augmented epistatic effects consisted of two epistatic effects of equal strength in opposite directions (200 replications).

*n*	*m*	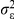	MSe	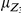	QTL_2_×QTL_3_							
					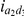	Power	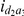	Power	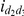	Power	Position_2_	Position_3_
Parameter values				0.50	1.50		-1.00		-1.50		50.00	50.00
200	5	4.00	9.868(0.969)	0.489(0.201)	2.397(0.281)	0.205	-2.342(0.353)	0.040	-3.292(0.286)	0.060	50.200(6.571)	49.550(6.597)
		1.00	6.303(0.622)	0.484(0.191)	2.055(0.296)	0.330	-1.933(0.200)	0.085	-2.811(0.424)	0.105	50.550(5.863)	49.500(5.559)
	10	4.00	4.946(0.502)	0.484(0.147)	1.879(0.288)	0.540	-1.681(0.161)	0.140	-2.429(0.272)	0.185	49.600(5.657)	49.950(4.860)
		1.00	3.224(0.350)	0.506(0.138)	1.656(0.280)	0.700	-1.412(0.173)	0.240	-2.079(0.282)	0.335	50.000(4.702)	50.300(4.243)
400	5	4.00	9.953(0.775)	0.490(0.155)	1.866(0.302)	0.535	-1.705(0.201)	0.095	-2.422(0.283)	0.205	50.650(5.589)	49.800(5.395)
		1.00	6.312(0.496)	0.511(0.126)	1.638(0.274)	0.780	-1.404(0.143)	0.275	-2.050(0.259)	0.390	49.950(4.860)	50.200(4.005)
	10	4.00	4.923(0.350)	0.501(0.121)	1.591(0.284)	0.910	-1.314(0.219)	0.405	-1.856(0.264)	0.490	49.950(4.312)	49.850(3.680)
		1.00	3.200(0.237)	0.493(0.089)	1.499(0.266)	0.995	-1.106(0.157)	0.725	-1.595(0.267)	0.825	49.900(3.006)	49.950(2.351)

* *n* denotes sample size; *m is* family replication number; and 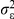 is residual variance for the phenotypic trait value 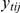.

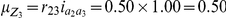
, see Model (7) for details.

### Experiment III

We simulated a large genome to explore the performance of the proposed method in real data analysis. The simulated genome was 1000.0 cM in total length and covered by 210 markers (10 chromosomes, each covered with twenty-one 5.0 cM equally spaced markers). Ten main-effect QTL and three pairs of interacted QTL, which totally explained ∼50% variation of L_1_, L_2_ and L_3_, were assumed (Tables 12 and 13). The environmental variance (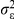), sample size and family replication number were set at 6.0, 500 and 10, respectively. The mapping results from 200 samples under the F_2_ metric model were presented in Table 12 for the main-effect QTL and Table 13 for the epistatic QTL. Results from Table 12 showed that all the augmented main effects were unbiasedly estimated with satisfactory powers; and most pure additive and dominance effects were also unbiasedly estimated with the exception of pure dominance effects for QTL_5_ and QTL_8_. The results from Table 13 demonstrated that with 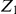 and 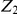 the augmented epistatic effects (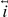 and 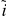) were well estimated when they consisted of two epistatic effects with same sign (QTL_4_ and QTL_7_, QTL_9_ and QTL_10_) and were poorly detected when they consisted of two epistatic effects of equal strength in opposite directions (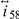 and 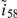 for QTL_5_ and QTL_8_); with 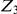 all the pure epistatic effects (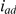, 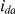 and 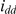) were well estimated, and no matter what signs they were; and all pure epistatic effects (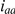, 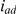, 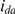 and 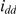) estimated in the second stage were unbiased except for 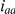 for QTL_5_ and QTL_8_ (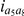). The failure of detecting 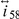 resulted in biased estimate for 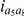, which further caused bad estimate for 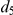 and 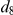. These results were similar to those in simulation experiments I and II. The time cost was ∼4.70h per sample on our person computer (CPU: Intel® CoreTM 2 DUO 3.0G, Memory: 2.0G).

**Table 12:** Simulated and estimated main-effect QTL position and effects for large genome data under the F_2_ metric model (200 replications).

MaineffectQTL	True parameter					Estimate at the first stage						Estimate at the second stage				
	Posi.(cM)	Pure main effects		Augmented main effects		*Z*_1_			*Z*_2_			*a*	Power	*d*	Power	Posi.
		*a*	*d*	*a* ^*^	*d* ^*^	*a* ^*^	Posi.	Power	*d* ^*^	Posi.	Power					
QTL_1_	30.00	-1.00	0.50	-1.00	0.50	-0.992(0.094)	30.000(0.709)	1.000	0.510(0.092)	28.453(6.726)	0.695	-0.992(0.094)	1.000	0.510(0.092)	0.695	29.463(2.878)
QTL_2_	75.00	1.00	-1.00	1.00	-1.00	0.987(0.098)	74.949(1.131)	0.980	-0.937(0.155)	75.003(1.642)	1.000	0.987(0.098)	0.980	-0.937(0.155)	1.000	74.997(1.119)
QTL_3_	150.00	0.70	0.00	0.70	0.00	0.677(0.096)	150.102(3.078)	0.980	.(.)	.(.)	.	0.677(0.096)	0.980			150.102(3.078)
QTL_4_	235.00	1.50	-1.00	1.00	-1.50	0.993(0.099)	235.029(0.797)	0.995	-1.468(0.107)	234.975(0.354)	1.000	1.482(0.155)	1.000	-1.006(0.263)	1.000	235.002(0.436)
QTL_5_	465.00	1.20	0.60	1.50	0.90	1.488(0.110)	465.000(0.000)	1.000	0.882(0.099)	465.189(1.426)	0.985	1.207(0.171)	1.000	0.207(0.367)	1.000	465.093(0.708)
QTL_6_	555.00	-0.50	1.00	-0.50	1.00	-0.500(0.086)	555.211(5.339)	0.910	0.976(0.108)	555.048(1.329)	0.995	-0.500(0.086)	0.910	0.976(0.108)	0.995	555.133(2.636)
QTL_7_	675.00	-1.00	1.50	-1.75	1.00	-1.744(0.096)	675.000(0.000)	1.000	0.993(0.112)	675.162(1.301)	0.995	-0.997(0.138)	1.000	1.450(0.272)	1.000	675.080(0.649)
QTL_8_	740.00	-0.70	1.30	-1.30	1.60	-1.295(0.097)	739.975(0.354)	1.000	1.584(0.105)	740.000(0.000)	1.000	-0.697(0.210)	1.000	0.922(0.361)	1.000	739.988(0.177)
QTL_9_	830.00	0.00	0.00	0.50	0.50	0.534(0.106)	829.632(5.402)	0.815	0.524(0.098)	829.588(6.104)	0.910	0.083(0.327)	0.900	0.021(0.516)	0.985	829.477(4.845)
QTL_10_	870.00	0.00	0.00	0.50	0.50	0.535(0.099)	869.859(4.750)	0.885	0.512(0.096)	870.322(6.115)	0.855	0.112(0.349)	0.955	-0.018(0.547)	0.990	869.987(4.063)

**Table 13:** Simulated and estimated epistatic QTL positions and effects for large genome data under the F_2_ metric model (200 replications).

EpistaticQTL	True parameter								Estimate at the first stage													
	Posi. A(cM)	Posi. B(cM)	Pure epistatic effects				Augmentedepistatic effects		Z1				Z2				Z3					
			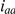	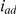	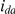	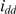	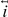	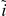	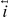	Posi. A	Posi.	Power	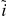	Posi. A	Posi. B	Power	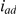	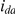	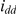	Posi. A	Posi. B	Power
QTL4×QTL7	235.00	675.00	1.00	1.50	1.00	1.50	2.50	2.50	2.501(0.287)	234.995(2.309)	675.025(1.543)	1.000	2.450(0.312)	234.922(2.102)	674.930(1.872)	0.980	1.528(0.342)	1.022(0.348)	1.577(0.485)	236.025(6.405)	675.550(4.854)	1.000
QTL5×QTL8	465.00	740.00	-0.60	1.20	-0.60	1.20	0.60	0.60	1.092(.)	475.000(.)	740.000(.)	0.005	1.078(0.173)	466.486(17.633)	739.324(18.603)	0.185	1.257(0.299)	-0.632(0.352)	1.424(0.575)	464.516(8.886)	740.591(6.100)	0.930
QTL9×QTL10	830.00	870.00	-1.00	-1.00	-1.00	-1.00	-2.00	-2.00	-1.971(0.275)	829.578(3.499)	870.361(3.836)	0.830	-1.935(0.337)	830.300(2.664)	870.287(4.207)	0.985	-1.223(0.478)	-1.187(0.532)	-1.270(0.554)	823.921(10.713)	875.612(10.098)	0.695

### Experiment IV

This simulation experiment was to consider the situation that QTL stands on the position in the marker interval. The three simulated QTL were placed at 45.0 (the middle of marker interval), 52.5 (the right of the sixth marker) and 47.5 cM (the left of the sixth marker), respectively. The number of individuals (*m*) for each TTC family was set at 5 and 10. The other settings were the same as those in the Experiment I. The results were shown in Table 14, Table 15, Table 16. The accuracies for the effects and the positions of QTL, as well as the empirical power, were satisfied but lower than those presented in Table 3, Table 4, Table 5; and the QTL effects were slightly underestimated because of the recombination between QTL and its adjacent marker.

**Table 14:** Results of mapping QTL of *Z*_1_ under F_2_ metric model while the simulated QTL were placed on the position in the marker intervals (200 replications).

*n*	*m*	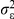	MSe	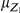	QTL_1_			QTL_2_			QTL_3_			QTL_2_×QTL_3_			
					*a_1_* *	Position	Power	*a_2_* *	Position	Power	*a_3_* *	Position	Power	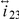	Position_2_	Position_3_	Power
Parameter values				199.25	1.50	45.00		1.50	52.50		-1.75	47.50		2.50	52.50	47.50	
200	5	4.00	3.103(0.372)	199.934(0.694)	1.382(0.199)	45.380(5.393)	0.970	1.423(0.210)	51.059(3.507)	0.995	-1.648(0.227)	49.096(3.064)	0.955	2.626(0.414)	52.674(6.217)	46.628(7.763)	0.430
		1.00	1.822(0.246)	199.535(0.505)	1.382(0.229)	45.209(4.495)	0.990	1.415(0.185)	50.901(2.590)	0.985	-1.649(0.203)	48.993(2.324)	1.000	2.367(0.323)	52.369(5.387)	47.588(6.719)	0.805
	10	4.00	1.696(0.212)	199.529(0.510)	1.374(0.230)	45.632(4.376)	0.980	1.438(0.186)	50.952(2.923)	1.000	-1.651(0.195)	49.151(2.174)	1.000	2.360(0.321)	52.184(7.483)	48.710(6.328)	0.815
		1.00	1.062(0.145)	199.353(0.241)	1.407(0.221)	45.281(3.372)	1.000	1.446(0.149)	51.105(2.159)	1.000	-1.698(0.132)	48.983(1.823)	1.000	2.328(0.281)	51.180(4.350)	48.610(4.512)	0.970
400	5	4.00	2.960(0.220)	199.401(0.333)	1.412(0.245)	45.133(3.907)	0.985	1.434(0.151)	50.673(1.819)	1.000	-1.653(0.191)	49.272(1.600)	1.000	2.319(0.321)	51.885(4.285)	48.303(5.823)	0.935
		1.00	1.743(0.142)	199.327(0.139)	1.462(0.159)	44.936(2.582)	1.000	1.449(0.128)	50.911(1.655)	1.000	-1.690(0.120)	49.059(1.454)	1.000	2.312(0.282)	51.512(3.547)	48.608(3.536)	0.985
	10	4.00	1.653(0.128)	199.349(0.149)	1.468(0.164)	44.879(2.565)	1.000	1.439(0.148)	50.761(1.395)	1.000	-1.697(0.136)	49.032(1.347)	1.000	2.263(0.301)	51.528(3.598)	49.020(2.967	0.985
		1.00	1.048(0.085)	199.315(0.129)	1.484(0.095)	44.978(1.871)	1.000	1.467(0.106)	51.103(1.353)	1.000	-1.716(0.086)	48.868(1.138)	1.000	2.315(0.283)	51.226(2.697)	48.429(3.104	0.995

* *n* denotes sample size; *m* is number of replications; and 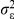 is residual variance for the phenotypic trait value 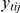.

** 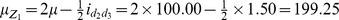, 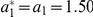, 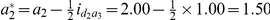, 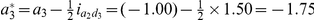 and 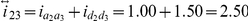, see model (3) for details.

**Table 15:** Results of mapping QTL of *Z*_2_ under F_2_ metric model while the simulated QTL were placed on the position in the marker intervals (200 replications).

*n*	*m*	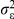	MSe	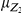	QTL_1_			QTL_2_			QTL_3_			QTL_2_×QTL_3_			
					*d_1_* *	Position	Power	*d_2_* *	Position	Power	*d_3_* *	Position	Power	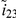	Position_2_	Position_3_	Power
Parameter values				2.50	1.50	45.00		-1.50	52.50		1.50	47.50		2.50	52.50	47.50	
200	5	4.00	2.918(0.329)	2.500(0.166)	1.354(0.196)	44.345(5.383)	0.970	-1.445(0.196)	51.141(3.880)	0.980	1.430(0.175)	48.471(3.585)	0.985	2.495(0.439)	52.555(7.982)	48.046(8.177)	0.755
		1.00	1.735(0.204)	2.488(0.120)	1.362(0.207)	44.709(4.701)	0.970	-1.392(0.193)	50.974(2.755)	0.985	1.399(0.198)	49.030(2.688)	0.990	2.358(0.351)	51.678(6.702)	47.335(5.861)	0.940
	10	4.00	1.630(0.187)	2.497(0.115)	1.378(0.250)	44.334(4.362)	0.990	-1.428(0.188)	51.032(2.771)	1.000	1.414(0.187)	48.730(3.069)	0.995	2.340(0.403)	52.220(7.249)	47.897(6.887)	0.965
		1.00	1.027(0.126)	2.500(0.091)	1.445(0.165)	45.383(3.277)	1.000	-1.430(0.142)	50.842(2.484)	1.000	1.448(0.115)	49.100(1.997)	1.000	2.311(0.350)	51.953(5.437)	48.031(4.709)	0.995
400	5	4.00	2.904(0.237)	2.513(0.118)	1.357(0.259)	45.455(4.525)	0.985	-1.435(0.174)	50.635(2.334)	1.000	1.408(0.209)	49.174(2.253)	1.000	2.303(0.372)	51.832(5.540)	48.400(6.380)	0.980
		1.00	1.692(0.132)	2.506(0.118)	1.461(0.151)	45.438(3.090)	1.000	-1.442(0.131)	50.800(1.519)	1.000	1.448(0.120)	49.086(1.730)	1.000	2.327(0.273)	51.056(3.449)	49.012(3.805)	0.990
	10	4.00	1.616(0.128)	2.504(0.085)	1.466(0.139)	45.303(2.551)	1.000	-1.439(0.146)	50.937(1.562)	1.000	1.450(0.127)	49.144(1.394)	1.000	2.310(0.322)	51.562(4.018)	48.482(3.988)	0.990
		1.00	1.007(0.089)	2.487(0.072)	1.488(0.080)	44.938(1.938)	1.000	-1.459(0.110)	50.962(1.240)	1.000	1.466(0.096)	49.047(1.250)	1.000	2.306(0.320)	51.389(4.439)	48.676(3.299)	0.995

* *n* denotes sample size; *m* is number of replications; and 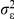 is residual variance for the phenotypic trait value 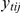.

** 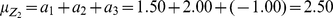, 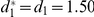, 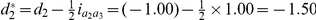, 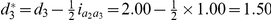 and 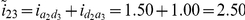, see Model (6) for details.

**Table 16:** Results of mapping QTL of *Z*_3_ under F_2_ metric model while the simulated QTL were placed on the position in the marker intervals (200 replications).

*n*	*m*	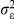	MSe	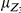	QTL_2_×QTL_3_							
					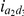	Power	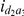	Power	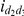	Power	Position_2_	Position_3_
Parameter values				0.50	1.50		1.00		1.50		52.50	47.50
200	5	4.00	9.856(1.065)	0.505(0.232)	2.479(0.319)	0.185	2.335(0.220)	0.070	3.248(0.256)	0.055	53.450(10.253)	45.950(9.139)
		1.00	6.352(0.637)	0.496(0.175)	2.017(0.237)	0.320	1.865(0.199)	0.105	2.786(0.322)	0.075	52.600(8.580)	46.600(7.598)
	10	4.00	4.949(0.514)	0.496(0.162)	1.839(0.237)	0.475	1.716(0.175)	0.115	2.406(0.291)	0.150	53.100(8.932)	47.000(7.569)
		1.00	3.220(0.325)	0.496(0.140)	1.610(0.251)	0.810	1.439(0.193)	0.255	1.995(0.254)	0.280	51.950(7.346)	48.050(6.073)
400	5	4.00	9.997(0.689)	0.493(0.160)	1.850(0.279)	0.465	1.704(0.189)	0.120	2.508(0.298)	0.095	53.650(8.517)	47.000(7.298)
		1.00	6.416(0.485)	0.495(0.130)	1.624(0.270)	0.730	1.400(0.156)	0.260	1.964(0.235)	0.270	52.600(6.963)	48.300(5.592)
	10	4.00	5.057(0.352)	0.499(0.119)	1.542(0.276)	0.865	1.293(0.188)	0.405	1.811(0.228)	0.425	52.350(6.495)	48.600(4.488)
		1.00	3.276(0.202)	0.505(0.089)	1.427(0.224)	0.985	1.128(0.147)	0.605	1.609(0.252)	0.635	51.450(5.342)	48.500(4.341)

* *n* denotes sample size; *m* is family replication number; and 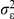 is residual variance for the phenotypic trait value 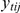.

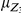
 = 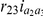 = 0.5×1.00 = 0.50, see Model (7) for details.

## Discussion

Compared to previous studies on the methodologies for the TTC, the method described here offers advantages over the previous approaches. First, with 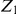 or 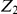 all augmented main and epistatic effects (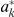, 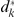, 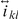 and 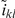) were included simultaneously in one genetic model and estimated together by the E-Bayes approach. Our simulation studies showed that these augmented effects could be estimated with very high power and precision when the component epistatic effects (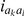 and 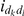 or 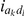 and 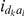) of 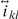 and 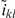 have the same direction (Tables 3, 4 and 13). Even though these epistatic effects have different signs, the new approach works well for augmented main-effect QTL parameters (Tables 9, 10 and 12).

Second, with 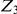 three pure epistatic effects (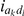, 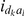 and 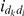) were estimated simultaneously in this study by two-dimensional genome scans. Although we attempted to use a full genetic model that included all the digenic epistatic effects for the estimation of all the epistatic effects under the framework of E-Bayes, it failed. The reasons are unclear. To date, there have been several approaches to detect the epistasis in the RIL-based TTC and NCIII designs, little is currently reported about the estimation of more than two epistatic effects in the TTC. Frascaroli et al. [16] and Li et al. [17] adopted the mixed linear model approach of Wang et al. [20] to detect 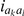 in the analyses of 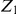 and 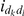 in the analyses of 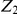; and Kusterer et al. [22] and Melchinger et al. [21] used two-way ANOVA on 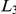 and 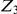 for the detection of 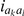 and 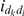, respectively. However, the two studies involved only one digenic epistatic effect. Although multiple interval mapping has been used to detect the augmented epistatic effects (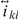 and 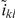) by Garcia et al. [34], the genetic design is NCIII and the estimate is a compound effect, not a pure epistatic effect. In addition, Reif et al. [24] proposed a two-step procedure to detect 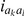 with particular two-segment NILs.

Finally, many main and epistatic effects can be estimated in an unambiguous and unbiased manner by our two-step approach. In the first step, the augmented main and epistatic effects (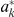,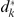,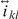 and 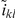) and three pure epistatic effects (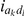, 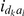 and 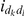) may be estimated in the separate analyses of 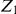, 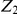 and 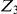. In the next step, all four pure epistatic effects (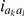, 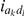, 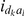 and 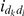) may be estimated by using the equation 
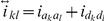
 and 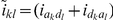 and pure additive and dominant effects may be further estimated by using the equations of 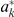 and 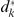. The simulation results show that the two-step approach works well (Tables 6, 12 and 13). However, the pure epistatic effects (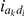, 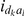 and 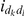) could not be detected with satisfactory statistical power when the sample size (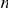) and family replication number (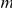) were low (Tables 5 and 11). Therefore, a large 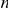 and 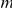 are needed for the detection of epistasis. To accommodate larger 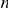, suitable field experimental designs, such as split-plot design [13], [16] and block in replication [35], are desired to control for environmental error.

The F_2_-based TTC design is superior to the F_2_ design for the detection of main-effect and epistatic QTL when there is a small sample size and a large residual variance (Tables 3, 4, 5 and 7), and is more powerful for estimating 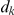, 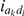 (or 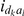) and especially 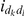 than the F_2:3_ design (Tables 4, 5 and 8). The new method may be extended to the TTC design derived from other base populations, such as RIL, BC and DH. This is because the genetic models for 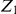, 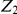 and 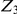 in these new TTC designs can be described in the same manner. In Tables S7, S8 and **Supporting Information S3** we only presented the expected genetic values and genetic variance for 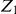, 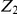 and 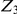 under both the F_2_ and the F_∞_ metric models in the RIL-based TTC design.

The proposed approach in this study differs from the previous methods of Kearsey et al. [12], Frascaroli et al. [16], Melchinger et al. [7], [21] and Li et al. [17]. First, the former derives the linear regression models for 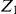, 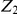 and 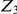 and the latter makes use of ANOVA. Thus, the precondition for the former is to derive the dummy variables for each genetic effects, whereas the precondition for the latter is to obtain the expectation and expected mean squares. In the expectation and expected mean squares, if one effect is confounded by another effect, these confounded effects may be estimated together. That is the augmented effect in the above ANOVA. If there are multicollinear relationships among dummy variables, the corresponding effects cannot be estimated. However, the effect combination is estimable. That is the augmented effect in the linear regression analysis. This can explain why we construct augmented effects. Second, we consider all the main-effect QTL and all the digenic interactions in one model of Z_1_ or Z_2_, all the augmented additive, dominance and epistatic effects have been rightly defined, and all the pure main and epistatic effects can be unbiasedly estimated. Although in the previous studies the augmented additive and dominant effects (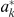 and 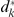) have been rightly defined and are clearly confounded by QTL × genetic background epistasis in the RIL-based TTC and NCIII designs [7], [21], [22], the augmented epistatic effects have been ignored. This neglect would result in a biased estimation for the augmented main effects, a larger residual variance and a lower power of QTL detection (Tables 3 and 4). In addition, with Z_3_ we can estimate three types of pure epistatic effects (*ad*, *da* and *dd*) using two-dimensional genome scans. This differs from Melchinger et al. [21], in which only *dd* epistasis can be obtained.

The F_2_ and F_∞_ are two main metrics that are adopted for populations derived from a cross between two inbred lines. The F_2_ metric is orthogonal for the F_2_ population when epistatic genes are under linkage equilibrium, whereas the F_∞_ metric is orthogonal for homozygous lines [28]–[30]. An orthogonal model implies that estimates of the genetic effects are consistent in a full and reduced model and is directly related to the partition of the genetic variance in the population. Using different models does not influence the detection of the main and epistatic QTL, but it does influence the estimation and interpretation of genetic effects [30]. Melchinger et al. [7], [21] and Kusterer et al. [13], [22] advocated the F_2_ metric in the RIL-based NCIII and TTC designs for three reasons: (1) it has the advantage that each variance component is proportional to the sum of the squares of the corresponding genetic effects and does not involve any other type of genetic effects that could obscure their interpretation; (2) epistatic interactions by two-way ANOVAs for pairs of marker loci using 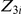 was just 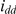; and (3) with digenic epistasis, midparent heterosis 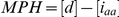 involves only 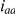 beside dominance effects, whereas under the F_∞_ metric MPH is additionally influenced by 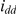. For F_2_-based TTC design, neither F_2_ nor F_∞_ metric models are orthogonal (**Supporting Information S2**). With the Z_1_ and Z_2_ the newly defined parameters (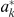, 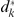, 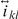 and 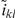) were all rightly identified and estimated by our full model methods under both metrics (Tables 3, 4, 12 and 13), and with Z_3_ the pure epistatic effects (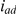, 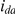, and 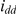) could also be detected and well estimated under both metrics when the sample size and number of family replications were large in our simulation studies (Tables 5, 11 and 13). The differences under the two metrics may be as follows: (1) the newly defined main effects and model means are different for the *Z*_1_ and *Z*_2_ under the two models; and (2) the F_2_ metric model seems to behave better than the F_∞_ metric model (higher power and precision) (data not shown).

The proposed approach in this study assumes that all the QTL stand on the markers. When marker density is high, all the QTL can be detected with a high power and precision. When marker density is sparse, the QTL effects are slightly underestimated because of the recombination between QTL and its adjacent marker. To solve the issue, some virtual marker (treated as missing data) may be inserted. At this time marker imputation techniques may be used.

The drawbacks for our method may lie in two aspects: (1) with 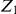 and 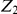 the augmented epistatic effects (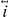 and 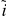) were poorly detected when their corresponding components have an equal strength in opposite directions (Tables 9, 10 and 13). This would result in biased estimate for pure *aa* epistatic effect, such as 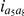 in Table 13, and further cause bad estimate for pure dominance effect, such as 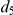 and 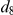 in Table 12; and (2) The estimation error for the pure main and epistatic effects using the two-step approach seemed to be a little large. This will be studied in the future.

## Supporting Information

Supporting Information S1 Statistical genetic models for mapping QTL in the TTC design under the F_∞_ metric model.
(DOC)

Supporting Information S2The expected genetic values of 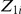, 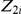 and 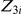 under the F_2_ and the F_∞_ metric models in the F_2_-based TTC design.(DOC)

Supporting Information S3The expected genetic values of the 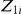, 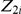 and 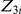 values under the F_∞_ and the F_2_ metric models in the RIL-based TTC design.(DOC)

Table S1 Genetic constitutions of the F_2_-based TTC family means *L*_1*i*_, *L*_2*i*_ and *L*_3*i*_.
(DOC)

Table S2 Expected genetic value of *L*_1*i*_ family under the F_2_ and the F_∞_ metric models in the F_2_-based TTC design.
(DOC)

Table S3 Expected genetic value of *L*_2*i*_ family under the F_2_ and the F_∞_ metric models in the F_2_-based TTC design.
(DOC)

Table S4 Expected genetic value of *L*_3*i*_ family under the F_2_ and the F_∞_ metric models in the F_2_-based TTC design.
(DOC)

Table S5Expected genetic values of 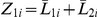, 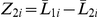 and 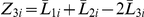 under the F_2_ metric model in the F_2_-based TTC design.(DOC)

Table S6Expected genetic values of 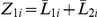, 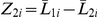 and 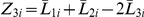 under the F_∞_ metric model in the F_2_-based TTC design.(DOC)

Table S7Expected genetic values of 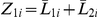,
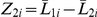
and 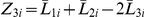 under the F_2_ metric model in the RIL-based TTC design.(DOC)

Table S8Expected genetic values of 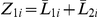, 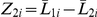 and 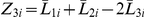 under the F_∞_ metric model in the RIL-based TTC design.(DOC)

## References

[1] Carlborg Ö, Haley CS (2004). Epistasis: too often neglected in complex trait studies.. Nat Rev Genet.

[2] Moore JH, Williams SM (2005). Traversing the conceptual divide between biological and statistical epistasis: systems biology and a more modern synthesis.. BioEssays.

[3] Jinks JL, Jones RM (1958). Estimation of the components of heterosis.. Genetics.

[4] Yu SB, Li JX, Xu CG, Tan YF, Gao YJ (1997). Importance of epistasis as the genetic basis of heterosis in an elite rice hybrid.. Proc Natl Acad Sci USA.

[5] Lippman ZB, Zamir D (2006). Heterosis: revisiting the magic.. Trends in Genetics.

[6] Melchinger AE, Piepho HP, Utz HF, Muminovi J, Wegenast T (2007). Genetic basis of heterosis for growth-related traits in Arabidopsis investigated by testcross progenies of near-isogenic lines reveals a significant role of epistasis.. Genetics.

[7] Melchinger AE, Utz HF, Piepho HP, Zeng ZB, Schön CC (2007). The role of epistasis in the manifestation of heterosis: a systems-oriented approach.. Genetics.

[8] Wright S (1980). Genic and organismic selection.. Evolution.

[9] Carson HL, Templeton AR (1984). Genetic revolutions in relation to speciation phenomena: the founding of new populations.. Annu Rev Ecol Syst.

[10] Kearsey MJ, Jinks JL (1968). A general method of detecting additive, dominance and epistatic variation for metrical traits. I. Theory.. Heredity.

[11] Kearsey MJ, Pooni HS (1996). The genetical analysis of quantitative traits..

[12] Kearsey MJ, Pooni HS, Syed NH (2003). Genetics of quantitative traits in *Arabidopsis thaliana*.. Heredity.

[13] Kusterer B, Muminovic J, Utz HF, Piepho HP, Barth S (2007). Analysis of a triple testcross design with recombinant inbred lines reveals a significant role of epistasis in heterosis for biomass-related traits in Arabidopsis.. Genetics.

[14] Jinks JL, Perkins JM (1970). A general method for the detection of additive, dominance and epistatic components of variation III. F_2_ and backcross populations.. Heredity.

[15] Perkins JM, Jinks JL (1970). Detection and estimation of genotype-environmental, linkage and epistatic components of variation for a metrical trait.. Heredity.

[16] Frascaroli E, Canè MA, Landi P, Pea G, Giabfranceschi L (2007). Classical genetic and quantitative trait loci analyses of heterosis in a maize hybrid between two elite inbred lines.. Genetics.

[17] Li L, Lu K, Chen Z, Mu T, Hu Z (2008). Dominance, overdominance and epistasis condition the heterosis in two heterotic rice hybrids.. Genetics.

[18] Kearsey MJ, Hyne V (1994). QTL analysis: a simple ‘marker-regression’ approach.. Theor Appl Genet.

[19] Zeng ZB (1994). Precision mapping of quantitative trait loci.. Genetics.

[20] Wang DL, Zhu J, Li ZK, Paterson AH (1999). Mapping QTL with epistatic effects and QTL × environment interactions by mixed model approaches.. Theor Appl Genet.

[21] Melchinger AE, Utz HF, Schön CC (2008). Genetic expectations of quantitative trait loci main and interaction effects obtained with the triple testcross design and their relevance for the analysis of heterosis.. Genetics.

[22] Kusterer B, Piepho HP, Utz HF, Schön CC, Muminovic J (2007). Heterosis for biomass-related traits in Arabidopsis investigated by quantitative trait loci analysis of the triple testcross design with recombinant inbred lines.. Genetics.

[23] Jiang C, Zeng ZB (1995). Multiple trait analysis of genetic mapping for quantitative trait loci.. Genetics.

[24] Reif JC, Kusterer B, Piepho HP, Meyer RC, Altman T (2009). Unraveling epistasis with triple testcross progenies of near-isogenic lines.. Genetics.

[25] Zhu C, Zhang R (2007). Efficiency of triple test cross for detecting epistasis with marker information.. Heredity.

[26] Wang XF, Song W, Yang ZF, Wang YM, Tang ZX (2009). Improved genetic mapping of endosperm traits using NCIII and TTC designs.. Journal of Heredity.

[27] Lander ES, Botstein D (1989). Mapping Mendelian factors underlying quantitative traits using RFLP linkage maps.. Genetics.

[28] Kao CH, Zeng ZB (2002). Modeling epistasis of quantitative trait loci using Cockerham's model.. Genetics.

[29] Yang RC (2004). Epistasis of quantitative trait loci under different gene action models.. Genetics.

[30] Zeng ZB, Wang T, Zou W (2005). Modeling quantitative trait loci and interpretation of models.. Genetics.

[31] Zhang YM, Xu S (2005). A penalized maximum likelihood method for estimating epistatic effects of QTL.. Heredity.

[32] Xu S (2007). An empirical Bayes method for estimating epistatic effects of quantitative trait loci.. Biometrics.

[33] Xu S (2010). An expectation–maximization algorithm for the Lasso estimation of quantitative trait locus effects.. Heredity.

[34] Garcia AAF, Wang S, Melchinger AE, Zeng ZB (2008). Quantitative trait loci mapping and the genetic basis of heterosis in maize and rice.. Genetics.

[35] Cockerham CC, Zeng ZB (1996). Design III with marker loci.. Genetics.

