# Supplementary material for: A Complete Solution for Dissecting Pure Main and Epistatic Effects of QTL in Triple Testcross Design
Source: PLoS One. 2011 Sep 19;6(9):e24575. doi: 10.1371/journal.pone.0024575 (PMC3176238; doi:10.1371/journal.pone.0024575)
Supplement: Supporting Information S1 — Statistical genetic models for mapping QTL in the TTC design under the F∞ metric model. (DOC) [file pone.0024575.s001.doc]

**Supporting Information S1. Statistical genetic models for mapping QTL in the TTC design under the F_∞_ metric model.**

According to the expected genetic values of $Z_{1i}$, $Z_{2i}$ and $Z_{3i}$ under the F_∞_ metric model (Table S6), the phenotypic value of $Z_{1i}$ can be described as:

$Z_{1i}=2\mu+x_{\text{a}_{1}i}a_{1}+d_{1}+x_{\text{a}_{2}i}a_{2}+d_{2}+x_{\text{a}_{1}\text{a}_{2}i}i_{a_{1}a_{2}}+x_{\text{a}_{1}\text{d}_{2}i}i_{a_{1}d_{2}}+x_{\text{d}_{1}\text{a}_{2}i}i_{d_{1}a_{2}}+x_{\text{d}_{1}\text{d}_{2}i}i_{d_{1}d_{2}}+e_{1i}$ (S1)

where $\mu$ is the mean genotypic values of four homozygotes; $a_{1}$ and $d_{1}$ are additive and dominance effects of QTL A; $a_{2}$ and $d_{2}$ are additive and dominance effects of QTL B; $i_{a_{1}a_{2}}$, $i_{a_{1}d_{2}}$, $i_{d_{1}a_{2}}$ and $i_{d_{1}d_{2}}$ are additive × additive, additive × dominance, dominance × additive and dominance × dominance epistatic effects (See Appendix A for details); $x_{\text{a}_{1}i}$, $x_{\text{a}_{2}i}$, $x_{\text{a}_{1}\text{a}_{2}i}$, $x_{\text{a}_{1}\text{d}_{2}i}$, $x_{\text{d}_{1}\text{a}_{2}i}$ and $x_{\text{d}_{1}\text{d}_{2}i}$ are determined by the genotype of the *i*th F_2_ plant (Table S6); and $e_{1i}$ is the residual error with an $N(0,\sigma_{1}^{2})$ distribution. According to the results in Table S6, there are $x_{\text{a}_{1}\text{a}_{2}i}=x_{\text{d}_{1}\text{d}_{2}i}$ and $x_{\text{a}_{1}\text{d}_{2}i}=-x_{\text{d}_{1}\text{a}_{2}i}=$ . To solve the genetic parameters, model (S1) must be reduced to:

$Z_{1i}=\mu_{Z_{1}}+x_{\text{a}_{1}i}a_{1}^{*}+x_{\text{a}_{2}i}a_{2}^{*}+x_{\overleftrightarrow{\text{i}}_{12}i}\overleftrightarrow{i}_{12}+e_{1i}$ (S2)

where $\mu_{Z_{1}}=2\mu+d_{1}+d_{2}$, , , $\overleftrightarrow{i}_{12}=(i_{a_{1}a_{2}}+i_{d_{1}d_{2}})$ and $x_{\overleftrightarrow{\text{i}}_{12}i}=x_{\text{a}_{1}\text{a}_{2}i}$ $=x_{\text{d}_{1}\text{d}_{2}i}$.

If the quantitative trait was controlled by $q$ QTL, model (S2) should be extended to:

$Z_{1i}=\mu_{Z_{1}}+\sum_{k=1}^{q} x_{a_{k}i}a_{k}^{*}+\sum_{k=1}^{q-1} \sum_{l=k+1}^{q} x_{\overleftrightarrow{i}_{kl}i}\overleftrightarrow{i}_{kl}+e_{1i}$ (S3)

where $\mu_{Z_{1}}=2\mu+\sum_{k=1}^{q} d_{k}$ is model mean; the augmented additive effect of QTL $k$, which equals to the pure additive effect of QTL $k$ ($a_{k}$) add half the sum of the difference between *ad* and *da* epistatic effects of QTL $k$ with all other QTL in the whole genome; $\overleftrightarrow{i}_{kl}=i_{a_{k}a_{l}}+i_{d_{k}d_{l}}$ is augmented epistatic effect between QTL $k$ and $l$, which estimates the compounded effect of *aa* and *dd* epistatic effects between QTL $k$ and $l$. Coefficients $x_{\text{a}_{\text{k}}i}$ and $x_{\overleftrightarrow{\text{i}}_{\text{kl}}i}$ are determined by genotypes of the *k*th and *l*th QTL (marker) for the *i*th F_2_ plant (Table 1). Note that the coefficients for the genotype $M_{k}m_{k}M_{l}m_{l}$ were integrated by the frequencies of $M_{k}M_{l}/m_{k}m_{l}$ and $M_{k}m_{l}/m_{k}M_{l}$. Model (S3) considers multiple QTL and all types of digenic epistasis and can be used to obtain $a_{k}^{*}$ and $\overleftrightarrow{i}_{kl}$, simultaneously. The augmented epistatic effects ($\overleftrightarrow{i}_{kl}$) are ignored in one-dimensional genome scans on $Z_{1i}$ by CIM [21], this would result in a bigger residual error and lower statistical power for detecting $a_{k}^{*}$.

In the same way, the phenotypic value of $Z_{2i}$ can be described as:

$Z_{2i}=a_{1}+u_{\text{d}_{1}i}d_{1}+a_{2}+u_{\text{d}_{2}i}d_{2}+u_{\text{a}_{1}\text{a}_{2}i}i_{a_{1}a_{2}}+u_{\text{a}_{1}\text{d}_{2}i}i_{a_{1}d_{2}}+u_{\text{d}_{1}\text{a}_{2}i}i_{d_{1}a_{2}}+u_{\text{d}_{1}\text{d}_{2}i}i_{d_{1}d_{2}}+e_{2i}$ (S4)

where $a_{1}$, $d_{1}$, $a_{2}$, $d_{2}$, $i_{a_{1}a_{2}}$, $i_{a_{1}d_{2}}$, $i_{d_{1}a_{2}}$ and $i_{d_{1}d_{2}}$ are same as those in model (S1); $u_{\text{d}_{1}i}$, $u_{\text{d}_{2}i}$, $u_{\text{a}_{1}\text{a}_{2}i}$, $u_{\text{a}_{1}\text{d}_{2}i}$, $u_{\text{d}_{1}\text{a}_{2}i}$ and $u_{\text{d}_{1}\text{d}_{2}i}$ are determined by the genotype of the *i*th F_2_ plant (Table S6), and $e_{2i}$ is the residual error with an $N(0,\sigma_{2}^{2})$ distribution. According to the results in Table S6, there are $u_{\text{a}_{1}\text{d}_{2}i}=u_{\text{d}_{1}\text{a}_{2}i}$ and . To solve the genetic parameters, model (S4) must be reduced to:

$Z_{2i}=\mu_{Z_{2}}+u_{\text{d}_{1}i}d_{1}^{*}+u_{\text{d}_{2}i}d_{2}^{*}+u_{\tilde{\text{i}}_{12}i}\tilde{i}_{12}+e_{2i}$ (S5)

where $\mu_{Z_{2}}=a_{1}+a_{2}$, , , $\tilde{i}_{12}=(i_{a_{1}d_{2}}+i_{d_{1}a_{2}})$ and $u_{\tilde{\text{i}}_{12}i}=u_{\text{a}_{1}\text{d}_{2}i}$ $=u_{\text{d}_{1}\text{a}_{2}i}$.

If the quantitative trait was controlled by $q$ QTL, model (S5) should be extended to:

$Z_{2i}=\mu_{Z_{2}}+\sum_{k=1}^{q} u_{\text{d}_{\text{k}}i}d_{k}^{*}+\sum_{k=1}^{q-1} \sum_{l=k+1}^{q} u_{\tilde{\text{i}}_{\text{kl}}i}\tilde{i}_{kl}+e_{2i}$ (S6)

where $\mu_{Z_{2}}=\sum_{k=1}^{q} a_{k}$ is model mean; is the augmented dominance effect of QTL $k$, which equals to the pure dominance effect of QTL $k$ ($d_{k}$) minus half the sum of the difference between *aa* and *dd* epistatic effects of QTL $k$ with all other QTL in the whole genome; $\tilde{i}_{kl}=i_{a_{k}d_{l}}+i_{d_{k}a_{l}}$ is augmented epistatic effect between QTL $k$ and $l$, which estimates the compounded effect of *ad* and *da* epistatic effects between QTL $k$ and $l$;. Coefficients $u_{\text{d}_{\text{k}}i}$ and $u_{\tilde{\text{i}}_{\text{kl}}i}$ are determined by the genotypes of the *k*th and *l*th QTL (marker) for the *i*th F_2_ plant (Table 1). Similarly to Model (S3), Model (S6) considers multiple QTL and all types of digenic epistasis and can be used to obtain $d_{k}^{*}$ and $\tilde{i}_{kl}$, simultaneously. The augmented epistatic effects ($\tilde{i}_{kl}$) are overlooked in one-dimensional genome scans on $Z_{2i}$ by CIM [21], this would also result in a bigger residual error and lower statistical power for detecting $d_{k}^{*}$.

Similarly, the phenotypic value of $Z_{3i}$ can be described as:

$Z_{3i}=ri_{a_{1}a_{2}}+v_{\text{a}_{1}\text{d}_{2}i}i_{a_{1}d_{2}}+v_{\text{d}_{1}\text{a}_{2}i}i_{d_{1}a_{2}}+v_{\text{d}_{1}\text{d}_{2}i}i_{d_{1}d_{2}}+e_{3i}=\mu_{Z_{3}}+v_{\text{a}_{1}\text{d}_{2}i}i_{a_{1}d_{2}}+v_{\text{d}_{1}\text{a}_{2}i}i_{d_{1}a_{2}}+v_{\text{d}_{1}\text{d}_{2}i}i_{d_{1}d_{2}}+e_{3i}$ (S7)

where $\mu_{Z_{3}}=ri_{a_{1}a_{2}}$; $r$ is the recombination fraction between the 1st and 2nd QTL; $v_{\text{a}_{1}\text{d}_{2}i}$, $v_{\text{d}_{1}\text{a}_{2}i}$ and $v_{\text{d}_{1}\text{d}_{2}i}$ are determined by the genotype of the *i*th F_2_ plant (Table S6; Table 1); and $e_{3i}$ is the residual error with an $N(0,\sigma_{3}^{2})$ distribution. According to Model (S7), three types of pure epistatic effect (*ad*, *da* and *dd*) can be obtained in the analysis of the F_2_-based TTC $Z_{3i}$ data with two-dimensional genome scans. This is differ from the result of Melchinger et al. [21], in which only *dd* epistasis can be obtained with two-way ANOVAs on RIL-based TTC $Z_{3i}$
