# Supplementary material for: A Complete Solution for Dissecting Pure Main and Epistatic Effects of QTL in Triple Testcross Design
Source: PLoS One. 2011 Sep 19;6(9):e24575. doi: 10.1371/journal.pone.0024575 (PMC3176238; doi:10.1371/journal.pone.0024575)
Supplement: Supporting Information S2 — The expected genetic values of , and under the F2 and the F∞ metric models in the F2-based TTC design. (DOC) [file pone.0024575.s002.doc]

**Supporting Information S2. The expected genetic values of $Z_{1i}$, $Z_{2i}$ and $Z_{3i}$ under the F_2_ and the F_∞_ metric models in the F_2_-based TTC design**

We assume that a quantitative trait is controlled by two QTL, A and B, each with two alleles ($A$, $a$ and $B$, $b$). The recombination fraction between A and B was $r$. The genotypes of the two inbred lines (P_1_ and P_2_) and their F_1_ are $AABB$, $aabb$ and $AaBb$, respectively. The F_1_ produces four types of gametes $AB$, $Ab$, $aB$ and $ab$ with frequencies , , and . The gametes from F_1_ are combined to produce F_2_ population with 10 type genotypes (Table S1). Note that the two double heterozygotes $AB/ab$ and $Ab/aB$, with different gametes constitutions under linkage disequilibrium, should be distinguished. Individuals from the F_2_ are cross to P_1_ (with gamete $AB$), P_2_ (with gamete $ab$) and F_1_ to produce *L*_1_, *L*_2_ and *L*_3_ families. The genetic constitutions of the three families are given in Table S1.

The genotypic values of the nine genotypes in *L*_1_*_i_*, *L*_2_*_i_* and *L*_3_*_i_* families under the F_2_ metric are defined as Kao and Zeng [28],

where $\mu$ is model mean (mean genotypic values of F_2_ population when $r=0.5$); $a_{1}$ is additive effect of QTL A, i.e. one-half of the difference in genotypic value between the two homozygote means of $AA$ ($AABB$, $AABb$ and $AAbb$) and $aa$ ($aaBB$, $aaBb$ and $aabb$); $d_{1}$ is dominance effect of QTL A, i.e. the departure in genotypic value of the heterozygote mean of $Aa$ ($AaBB$,$AaBb$, and $Aabb$ ) from the midpoint between the two homozygote means of $AA$ ($AABB$, $AABb$ and $AAbb$) and $aa$ ($aaBB$, $aaBb$ and $aabb$); $a_{2}$ and $d_{2}$ are additive and dominance effects of QTL B, with similar definitions to QTL A; $i_{a_{1}a_{2}}$, $i_{a_{1}d_{2}}$, $i_{d_{1}a_{2}}$ and $i_{d_{1}d_{2}}$ are additive × additive, additive × dominance, dominance × additive and dominance × dominance epistatic effects. While under the F_∞_ metric, genotypic values of the nine genotypes are defined as Kao and Zeng [28],

$$\left[\begin{matrix} G_{AABB} \\ G_{AABb} \\ G_{AAbb} \\ G_{AaBB} \\ G_{AaBb} \\ G_{Aabb} \\ G_{aaBB} \\ G_{aaBb} \\ G_{aabb} \end{matrix}\right]=\left[\begin{matrix} 1 & 1 & 0 & 1 & 0 & 1 & 0 & 0 & 0 \\ 1 & 1 & 0 & 0 & 1 & 0 & 1 & 0 & 0 \\ 1 & 1 & 0 & -1 & 0 & -1 & 0 & 0 & 0 \\ 1 & 0 & 1 & 1 & 0 & 0 & 0 & 1 & 0 \\ 1 & 0 & 1 & 0 & 1 & 0 & 0 & 0 & 1 \\ 1 & 0 & 1 & -1 & 0 & 0 & 0 & -1 & 0 \\ 1 & -1 & 0 & 1 & 0 & -1 & 0 & 0 & 0 \\ 1 & -1 & 0 & 0 & 1 & 0 & -1 & 0 & 0 \\ 1 & -1 & 0 & -1 & 0 & 1 & 0 & 0 & 0 \end{matrix}\right]\left[\begin{matrix} \mu\\ a_{1} \\ d_{1} \\ a_{2} \\ d_{2} \\ i_{a_{1}a_{2}} \\ i_{a_{1}d_{2}} \\ i_{d_{1}a_{2}} \\ i_{d_{1}d_{2}} \end{matrix}\right]$$

where $\mu$ is model mean (mean genotypic values of four homozygotes, $AABB$, $AAbb$, $aaBB$ and $aabb$); $a_{1}$ is additive effect of QTL A, i.e. average substitution effect of $a$ (in $aaBB$ and $aabb$) by $A$; $d_{1}$ is dominance effect of QTL A, i.e. the departure in genotypic value of the heterozygote mean of $Aa$ ($AaBB$ and $Aabb$) from the midpoint between the two homozygote means of $AA$ ($AABB$ and $AAbb$) and $aa$ ($aaBB$ and $aabb$); $a_{2}$ and $d_{2}$ may be defined similarly; and $i_{a_{1}a_{2}}$, $i_{a_{1}d_{2}}$, $i_{d_{1}a_{2}}$ and $i_{d_{1}d_{2}}$ are additive × additive, additive × dominance, dominance × additive and dominance × dominance epistatic effects. Using the definitions above, the expected genetic values of *L*_1_*_i_*, *L*_2_*_i_* and *L*_3_*_i_* families can be calculated and are listed in Tables S2, S3 and S4. With these expected values, the expected values of the F_2_-based $Z_{1i}=\bar{L}_{1i}+\bar{L}_{2i}$, $Z_{2i}=\bar{L}_{1i}-\bar{L}_{2i}$ and $Z_{3i}=\bar{L}_{1i}+\bar{L}_{2i}-2\bar{L}_{3i}$ values can be further calculated and are presented in Table S5 for the F_2_ metric model and Table S6 for the F_∞_ metric model.

According to Table S5, genetic variance between families on $Z_{1i}$, $Z_{2i}$ and $Z_{3i}$ under the F_2_-metric model are:

$$\begin{matrix} V_{\text{G}}(Z_{1i})=\frac{1}{2}(a_{1}^{2}+a_{2}^{2})+\frac{1}{4}r(2-5r+4r^{2})(i_{a_{1}a_{2}}^{2}+i_{d_{1}d_{2}}^{2})+\frac{1}{8}(i_{a_{1}d_{2}}^{2}+i_{d_{1}a_{2}}^{2}) \\ +(1-2r)a_{1}a_{2}-\frac{1}{2}(1-2r)(a_{1}i_{a_{1}d_{2}}+a_{2}i_{d_{1}a_{2}})-\frac{1}{2}(a_{1}i_{d_{1}a_{2}}+a_{2}i_{a_{1}d_{2}})+\frac{1}{2}r(2-5r+4r^{2})i_{a_{1}a_{2}}i_{d_{1}d_{2}}+\frac{1}{4}(1-2r)i_{a_{1}d_{2}}i_{d_{1}a_{2}} \end{matrix}$$

$$V_{\text{G}}(Z_{2i})=\frac{1}{2}(d_{1}^{2}+d_{2}^{2})+\frac{1}{2}(1-r)i_{a_{1}a_{2}}^{2}+\frac{1}{4}r(2-5r+4r^{2})(i_{a_{1}d_{2}}^{2}+i_{d_{1}a_{2}}^{2})+(1-2r)d_{1}d_{2}-(1-r)(d_{1}+d_{2})i_{a_{1}a_{2}}+\frac{1}{2}r(2-5r+4r^{2})i_{a_{1}d_{2}}i_{d_{1}a_{2}}$$

$$V_{\text{G}}(Z_{3i})=\frac{1}{2}r^{2}(i_{a_{1}d_{2}}^{2}+i_{d_{1}a_{2}}^{2})+r^{3}(2-5r+4r^{2})i_{d_{1}d_{2}}^{2}+r^{2}(1-2r)i_{a_{1}d_{2}}i_{d_{1}a_{2}}$$

Likewise, genetic variance of among F_2_ individuals and between F_2:3_ families under the F_2_-metric model are:

$$\begin{matrix} V_{\text{G}}(\text{F}_{2})=\frac{1}{2}(a_{1}^{2}+a_{2}^{2})+\frac{1}{4}(d_{1}^{2}+d_{2}^{2})+\frac{1}{4}i_{a_{1}a_{2}}^{2}+\frac{1}{8}(i_{a_{1}d_{2}}^{2}+i_{d_{1}a_{2}}^{2})+\frac{1}{16}[1-(1-2r)^{4}]i_{d_{1}d_{2}}^{2} \\ +(1-2r)a_{1}a_{2}+\frac{1}{2}(1-2r)^{2}d_{1}d_{2}-\frac{1}{2}(1-2r)(a_{1}i_{d_{1}a_{2}}+a_{2}i_{a_{1}d_{2}})-\frac{1}{2}(1-2r)^{2}(a_{1}i_{a_{1}d_{2}}+a_{2}i_{d_{1}a_{2}}) \\ -\frac{1}{2}(1-2r)(d_{1}+d_{2})i_{a_{1}a_{2}}+r(1-r)(1-2r)i_{a_{1}a_{2}}i_{d_{1}d_{2}}+\frac{1}{4}(1-2r)i_{a_{1}d_{2}}i_{d_{1}a_{2}} \end{matrix}$$

$$\begin{matrix} V_{\text{G}}(\bar{\text{F}}_{2:3})=\frac{1}{2}(a_{1}^{2}+a_{2}^{2})+\frac{1}{16}(d_{1}^{2}+d_{2}^{2})+\frac{1}{16}(1+20r-44r^{2}+32r^{3})i_{a_{1}a_{2}}^{2}+\frac{1}{8}[r^{2}+(1-r)^{2}](i_{a_{1}d_{2}}^{2}+i_{d_{1}a_{2}}^{2}) \\ +\frac{1}{32}[r^{2}+(1-r)^{2}]\{1+(1-2r)^{4}-2[r^{2}+(1-r)^{2}]^{3}\}i_{d_{1}d_{2}}^{2} \\ +(1-2r)a_{1}a_{2}+\frac{1}{8}(1-2r)^{2}d_{1}d_{2}-\frac{1}{2}[r^{2}+(1-r)^{2}](a_{1}i_{a_{1}d_{2}}+a_{2}i_{d_{1}a_{2}})-\frac{1}{2}(1-2r)(a_{1}i_{d_{1}a_{2}}+a_{2}i_{a_{1}d_{2}}) \\ -\frac{1}{8}(1-4r^{2})(d_{1}+d_{2})i_{a_{1}a_{2}}-\frac{1}{4}r(1-r)[r^{2}+(1-r)^{2}](d_{1}+d_{2})i_{d_{1}d_{2}}+\frac{1}{4}(1-2r)i_{a_{1}d_{2}}i_{d_{1}a_{2}}+\frac{1}{2}r(1-2r)(2-5r+8r^{2}-7r^{3}+2r^{4})i_{a_{1}a_{2}}i_{d_{1}d_{2}} \end{matrix}$$

According to Table S6, genetic variance between families on $Z_{1i}$, $Z_{2i}$ and $Z_{3i}$ under the F_∞_-metric model are:

$$V_{\text{G}}(Z_{1i})=\frac{1}{2}(a_{1}^{2}+a_{2}^{2})+\frac{1}{4}r(2-5r+4r^{2})(i_{a_{1}a_{2}}^{2}+i_{d_{1}d_{2}}^{2})+\frac{1}{2}r(i_{a_{1}d_{2}}^{2}+i_{d_{1}a_{2}}^{2})+(1-2r)a_{1}a_{2}+r(a_{1}-a_{2})(i_{a_{1}d_{2}}-i_{d_{1}a_{2}})+\frac{1}{2}r(2-5r+4r^{2})i_{a_{1}a_{2}}i_{d_{1}d_{2}}-ri_{a_{1}d_{2}}i_{d_{1}a_{2}}$$

$$\begin{matrix} V_{\text{G}}(Z_{2i})=\frac{1}{2}(d_{1}^{2}+d_{2}^{2})+\frac{1}{2}(1-r)(i_{a_{1}a_{2}}^{2}+i_{d_{1}d_{2}}^{2})+\frac{1}{4}r(2-5r+4r^{2})(i_{a_{1}d_{2}}^{2}+i_{d_{1}a_{2}}^{2}) \\ +(1-2r)d_{1}d_{2}-(1-r)(d_{1}+d_{2})(i_{a_{1}a_{2}}-i_{d_{1}d_{2}})-(1-r)i_{a_{1}a_{2}}i_{d_{1}d_{2}}+\frac{1}{2}r(2-5r+4r^{2})i_{a_{1}d_{2}}i_{d_{1}a_{2}} \end{matrix}$$

$$V_{\text{G}}(Z_{3i})=\frac{1}{2}r^{2}(i_{a_{1}d_{2}}^{2}+i_{d_{1}a_{2}}^{2})+r^{3}(2-5r+4r^{2})i_{d_{1}d_{2}}^{2}+r^{2}(1-2r)i_{a_{1}d_{2}}i_{d_{1}a_{2}}$$

Likewise, genetic variance of among F_2_ individuals and between F_2:3_ families under the F_∞_-metric model are:

$$\begin{matrix} V_{\text{G}}(\text{F}_{2})=\frac{1}{2}(a_{1}^{2}+a_{2}^{2})+\frac{1}{4}(d_{1}^{2}+d_{2}^{2})+\frac{1}{4}i_{a_{1}a_{2}}^{2}+r(1-r)(i_{a_{1}d_{2}}^{2}+i_{d_{1}a_{2}}^{2})+[\frac{1}{4}-r^{2}(1-r)^{2}]i_{d_{1}d_{2}}^{2} \\ +(1-2r)a_{1}a_{2}+[-\frac{1}{2}+r^{2}+(1-r)^{2}]d_{1}d_{2}+2r(1-r)(a_{1}i_{a_{1}d_{2}}+a_{2}i_{d_{1}a_{2}}) \\ -\frac{1}{2}(1-2r)(d_{1}+d_{2})i_{a_{1}a_{2}}+\frac{1}{2}[r^{2}+(1-r)^{2}](d_{1}+d_{2})i_{d_{1}d_{2}}-\frac{1}{2}(1-2r)[r^{2}+(1-r)^{2}]i_{a_{1}a_{2}}i_{d_{1}d_{2}} \end{matrix}$$

$$\begin{matrix} V_{\text{G}}(\bar{\text{F}}_{2:3})=\frac{1}{2}(a_{1}^{2}+a_{2}^{2})+\frac{1}{16}(d_{1}^{2}+d_{2}^{2})+\frac{1}{16}(1+20r-44r^{2}+32r^{3})i_{a_{1}a_{2}}^{2}+\frac{1}{4}r(1-r)(i_{a_{1}d_{2}}^{2}+i_{d_{1}a_{2}}^{2})+\frac{1}{16}(1+2r-2r^{2})[r^{2}+(1-r)^{2}]^{3}i_{d_{1}d_{2}}^{2} \\ +(1-2r)a_{1}a_{2}+\frac{1}{8}(1-2r)^{2}d_{1}d_{2}+r(1-r)(a_{1}i_{a_{1}d_{2}}+a_{2}i_{d_{1}a_{2}})-\frac{1}{8}(1-4r^{2})(d_{1}+d_{2})i_{a_{1}a_{2}}+\frac{1}{8}[r^{2}+(1-r)^{2}]^{2}(d_{1}+d_{2})i_{d_{1}d_{2}} \\ +\frac{1}{8}(1-2r)[r^{2}+(1-r)^{2}][-1+2r(1-2r)(2-r)]i_{a_{1}a_{2}}i_{d_{1}d_{2}} \end{matrix}$$
