# Supplementary material for: A Complete Solution for Dissecting Pure Main and Epistatic Effects of QTL in Triple Testcross Design
Source: PLoS One. 2011 Sep 19;6(9):e24575. doi: 10.1371/journal.pone.0024575 (PMC3176238; doi:10.1371/journal.pone.0024575)
Supplement: Supporting Information S3 — The expected genetic values of the , and values under the F∞ and the F2 metric models in the RIL-based TTC design. (DOC) [file pone.0024575.s003.doc]

**Supporting Information S3. The expected genetic values of the $Z_{1i}$, $Z_{2i}$ and $Z_{3i}$ values under the F_∞_ and the F_2_ metric models in the RIL-based TTC design**

The genotype symbols and assignments are following the assumptions in Appendix A. In the RIL there are only four homozygotes, $AABB$, $AAbb$, $aaBB$ and $aabb$. The four genotypes have frequencies , , and , respectively, where $R=2r/(1+2r)$. We simply copied the expected genetic values of $Z_{1i}$, $Z_{2i}$ and $Z_{3i}$ in Table S5 and Table S6 and presented them into Tables S7 and S8.

According to Table S7, genetic variance between families on $Z_{1i}$, $Z_{2i}$ and $Z_{3i}$ under the F_2_-metric model are:

$$\begin{matrix} V_{\text{G}}(Z_{1i})_{\text{RIL}}=(a_{1}^{2}+a_{2}^{2})+R(1-R)(i_{a_{1}a_{2}}^{2}+i_{d_{1}d_{2}}^{2})+\frac{1}{4}(i_{a_{1}d_{2}}^{2}+i_{d_{1}a_{2}}^{2}) \\ +2(1-2R)a_{1}a_{2}-(1-2R)(a_{1}i_{a_{1}d_{2}}+a_{2}i_{d_{1}a_{2}})-(a_{1}i_{d_{1}a_{2}}+a_{2}i_{a_{1}d_{2}})+2R(1-R)i_{a_{1}a_{2}}i_{d_{1}d_{2}}+\frac{1}{2}(1-2R)i_{a_{1}d_{2}}i_{d_{1}a_{2}} \end{matrix}$$

$$V_{\text{G}}(Z_{2i})_{\text{RIL}}=(d_{1}^{2}+d_{2}^{2})+(1-R)i_{a_{1}a_{2}}^{2}+R(1-R)(i_{a_{1}d_{2}}^{2}+i_{d_{1}a_{2}}^{2})+2(1-2R)d_{1}d_{2}-2(1-R)(d_{1}+d_{2})i_{a_{1}a_{2}}+2R(1-R)i_{a_{1}d_{2}}i_{d_{1}a_{2}}$$

$$V_{\text{G}}(Z_{3i})_{\text{RIL}}=r^{2}(i_{a_{1}d_{2}}^{2}+i_{d_{1}a_{2}}^{2})+4R(1-R)r^{2}i_{d_{1}d_{2}}^{2}+2(1-2R)r^{2}i_{a_{1}d_{2}}i_{d_{1}a_{2}}$$

According to Table S8, genetic variance between families on $Z_{1i}$, $Z_{2i}$ and $Z_{3i}$ under the F_∞_-metric model are:

$$\begin{matrix} V_{\text{G}}(Z_{1i}\text{)}_{\text{RIL}}=(a_{1}^{2}+a_{2}^{2})+R(1-R)(i_{a_{1}a_{2}}^{2}+i_{d_{1}d_{2}}^{2})+R(i_{a_{1}d_{2}}^{2}+i_{d_{1}a_{2}}^{2}) \\ +2(1-2R)a_{1}a_{2}+2R(a_{1}-a_{2})(i_{a_{1}d_{2}}-i_{d_{1}a_{2}})+2R(1-R)i_{a_{1}a_{2}}i_{d_{1}d_{2}}-2Ri_{a_{1}d_{2}}i_{d_{1}a_{2}} \end{matrix}$$

$$\begin{matrix} V_{\text{G}}(Z_{2i})_{\text{RIL}}=(d_{1}^{2}+d_{2}^{2})+(1-R)(i_{a_{1}a_{2}}^{2}+i_{d_{1}d_{2}}^{2})+R(1-R)(i_{a_{1}d_{2}}^{2}+i_{d_{1}a_{2}}^{2}) \\ +2(1-2R)d_{1}d_{2}-2(1-R)(d_{1}+d_{2})(i_{a_{1}a_{2}}-i_{d_{1}d_{2}})-2(1-R)i_{a_{1}a_{2}}i_{d_{1}d_{2}}+2R(1-R)i_{a_{1}d_{2}}i_{d_{1}a_{2}} \end{matrix}$$

$$V_{\text{G}}(Z_{3i})_{\text{RIL}}=r^{2}(i_{a_{1}d_{2}}^{2}+i_{d_{1}a_{2}}^{2})+4R(1-R)r^{2}i_{d_{1}d_{2}}^{2}+2(1-2R)r^{2}i_{a_{1}d_{2}}i_{d_{1}a_{2}}$$
