# Supplementary material for: A Complete Solution for Dissecting Pure Main and Epistatic Effects of QTL in Triple Testcross Design
Source: PLoS One. 2011 Sep 19;6(9):e24575. doi: 10.1371/journal.pone.0024575 (PMC3176238; doi:10.1371/journal.pone.0024575)
Supplement: Table S1 — Genetic constitutions of the F2-based TTC family means L 1i, L 2i and L 3i. (DOC) [file pone.0024575.s004.doc]

**Table S1**

**Genetic constitutions of the F_2_-based TTC family means *L*_1_*_i_*, *L*_2_*_i_* and *L*_3_*_i_***

| Genotype of  F_2_ plant | Frequency | Genotypes in L_1_*_i_* (L_2_*_i_*) family | | | |  | Genotypes in L_3_*_i_* family | | | | | | | | |
| --- | --- | --- | --- | --- | --- | --- | --- | --- | --- | --- | --- | --- | --- | --- | --- |
|  |  | $AABB$ ($AaBb$) | $AABb$ ($Aabb$) | $AaBB$ ($aaBb$) | $AaBb$ ($aabb$) |  | $AABB$ | $AABb$ | $AAbb$ | $AaBB$ | $AaBb$ | $Aabb$ | $aaBB$ | $aaBb$ | $aabb$ |
| $\frac{\begin{matrix} A & B \end{matrix}}{\begin{matrix} A & B \end{matrix}}$ | $\frac{1}{4}(1-r)^{2}$ | $1$ | $0$ | $0$ | $0$ |  | $\frac{1}{2}(1-r)$ | $\frac{1}{2}r$ | $0$ | $\frac{1}{2}r$ | $\frac{1}{2}(1-r)$ | $0$ | $0$ | $0$ | $0$ |
| $\frac{\begin{matrix} A & B \end{matrix}}{\begin{matrix} A & b \end{matrix}}$ | $\frac{1}{2}r(1-r)$ | $\frac{1}{2}$ | $\frac{1}{2}$ | $0$ | $0$ |  | $\frac{1}{4}(1-r)$ | $\frac{1}{4}$ | $\frac{1}{4}r$ | $\frac{1}{4}r$ | $\frac{1}{4}$ | $\frac{1}{4}(1-r)$ | $0$ | $0$ | $0$ |
| $\frac{\begin{matrix} A & b \end{matrix}}{\begin{matrix} A & b \end{matrix}}$ | $\frac{1}{4}r^{2}$ | $0$ | $1$ | $0$ | $0$ |  | $0$ | $\frac{1}{2}(1-r)$ | $\frac{1}{2}r$ | $0$ | $\frac{1}{2}r$ | $\frac{1}{2}(1-r)$ | $0$ | $0$ | $0$ |
| $\frac{\begin{matrix} A & B \end{matrix}}{\begin{matrix} a & B \end{matrix}}$ | $\frac{1}{2}r(1-r)$ | $\frac{1}{2}$ | $0$ | $\frac{1}{2}$ | $0$ |  | $\frac{1}{4}(1-r)$ | $\frac{1}{4}r$ | $0$ | $\frac{1}{4}$ | $\frac{1}{4}$ | $0$ | $\frac{1}{4}r$ | $\frac{1}{4}(1-r)$ | $0$ |
| $\frac{\begin{matrix} A & B \end{matrix}}{\begin{matrix} a & b \end{matrix}}$ | $\frac{1}{2}(1-r)^{2}$ | $\frac{1}{2}(1-r)$ | $\frac{1}{2}r$ | $\frac{1}{2}r$ | $\frac{1}{2}(1-r)$ |  | $\frac{1}{4}(1-r)^{2}$ | $\frac{1}{2}r(1-r)$ | $\frac{1}{4}r^{2}$ | $\frac{1}{2}r(1-r)$ | $\frac{1}{2}[r^{2}+(1-r)^{2}]$ | $\frac{1}{2}r(1-r)$ | $\frac{1}{4}r^{2}$ | $\frac{1}{2}r(1-r)$ | $\frac{1}{4}(1-r)^{2}$ |
| $\frac{\begin{matrix} A & b \end{matrix}}{\begin{matrix} a & B \end{matrix}}$ | $\frac{1}{2}r^{2}$ | $\frac{1}{2}r$ | $\frac{1}{2}(1-r)$ | $\frac{1}{2}(1-r)$ | $\frac{1}{2}r$ |  | $\frac{1}{4}r(1-r)$ | $\frac{1}{4}[r^{2}+(1-r)^{2}]$ | $\frac{1}{4}r(1-r)$ | $\frac{1}{4}[r^{2}+(1-r)^{2}]$ | $r(1-r)$ | $\frac{1}{4}[r^{2}+(1-r)^{2}]$ | $\frac{1}{4}r(1-r)$ | $\frac{1}{4}[r^{2}+(1-r)^{2}]$ | $\frac{1}{4}r(1-r)$ |
| $\frac{\begin{matrix} A & b \end{matrix}}{\begin{matrix} a & b \end{matrix}}$ | $\frac{1}{2}r(1-r)$ | $0$ | $\frac{1}{2}$ | $0$ | $\frac{1}{2}$ |  | $0$ | $\frac{1}{4}(1-r)$ | $\frac{1}{4}r$ | $0$ | $\frac{1}{4}$ | $\frac{1}{4}$ | $0$ | $\frac{1}{4}r$ | $\frac{1}{4}(1-r)$ |
| $\frac{\begin{matrix} a & B \end{matrix}}{\begin{matrix} a & B \end{matrix}}$ | $\frac{1}{4}r^{2}$ | $0$ | $0$ | $1$ | $0$ |  | $0$ | $0$ | $0$ | $\frac{1}{2}(1-r)$ | $\frac{1}{2}r$ | $0$ | $\frac{1}{2}r$ | $\frac{1}{2}(1-r)$ | $0$ |
| $\frac{\begin{matrix} a & B \end{matrix}}{\begin{matrix} a & b \end{matrix}}$ | $\frac{1}{2}r(1-r)$ | $0$ | $0$ | $\frac{1}{2}$ | $\frac{1}{2}$ |  | $0$ | $0$ | $0$ | $\frac{1}{4}(1-r)$ | $\frac{1}{4}$ | $\frac{1}{4}r$ | $\frac{1}{4}r$ | $\frac{1}{4}$ | $\frac{1}{4}(1-r)$ |
| $\frac{\begin{matrix} a & b \end{matrix}}{\begin{matrix} a & b \end{matrix}}$ | $\frac{1}{4}(1-r)^{2}$ | $0$ | $0$ | $0$ | $1$ |  | $0$ | $0$ | $0$ | $0$ | $\frac{1}{2}(1-r)$ | $\frac{1}{2}r$ | $0$ | $\frac{1}{2}r$ | $\frac{1}{2}(1-r)$ |
