# Supplementary material for: A Complete Solution for Dissecting Pure Main and Epistatic Effects of QTL in Triple Testcross Design
Source: PLoS One. 2011 Sep 19;6(9):e24575. doi: 10.1371/journal.pone.0024575 (PMC3176238; doi:10.1371/journal.pone.0024575)
Supplement: Table S4 — Expected genetic value of L 3i family under the F2 and the F∞ metric models in the F2-based TTC design. (DOC) [file pone.0024575.s007.doc]

**Table S4 Expected genetic value of *L*_3_*_i_* family under the F_2_ and the F_∞_ metric models in the F_2_-based TTC design**

| Genotype of  F_2_ plant | F_2_ metric model | | | | | | | | |  | F_∞_ metric model | | | | | | | | |
| --- | --- | --- | --- | --- | --- | --- | --- | --- | --- | --- | --- | --- | --- | --- | --- | --- | --- | --- | --- |
|  | $\mu$ | $a_{1}$ | $d_{1}$ | $a_{2}$ | $d_{2}$ | $i_{a_{1}a_{2}}$ | $i_{a_{1}d_{2}}$ | $i_{d_{1}a_{2}}$ | $i_{d_{1}d_{2}}$ |  | $\mu$ | $a_{1}$ | $d_{1}$ | $a_{2}$ | $d_{2}$ | $i_{a_{1}a_{2}}$ | $i_{a_{1}d_{2}}$ | $i_{d_{1}a_{2}}$ | $i_{d_{1}d_{2}}$ |
| $\frac{\begin{matrix} A & B \end{matrix}}{\begin{matrix} A & B \end{matrix}}$ | $1$ | $\frac{1}{2}$ | $0$ | $\frac{1}{2}$ | $0$ | $\frac{1}{2}(1-r)$ | $-\frac{1}{4}(1-2r)$ | $-\frac{1}{4}(1-2r)$ | $\frac{1}{4}(1-2r)$ |  | $1$ | $\frac{1}{2}$ | $\frac{1}{2}$ | $\frac{1}{2}$ | $\frac{1}{2}$ | $\frac{1}{2}(1-r)$ | $\frac{1}{2}r$ | $\frac{1}{2}r$ | $\frac{1}{2}(1-r)$ |
| $\frac{\begin{matrix} A & B \end{matrix}}{\begin{matrix} A & b \end{matrix}}$ | $1$ | $\frac{1}{2}$ | $0$ | $0$ | $0$ | $\frac{1}{4}(1-2r)$ | $0$ | $-\frac{1}{4}(1-2r)$ | $0$ |  | $1$ | $\frac{1}{2}$ | $\frac{1}{2}$ | $0$ | $\frac{1}{2}$ | $\frac{1}{4}(1-2r)$ | $\frac{1}{4}$ | $-\frac{1}{4}(1-2r)$ | $\frac{1}{4}$ |
| $\frac{\begin{matrix} A & b \end{matrix}}{\begin{matrix} A & b \end{matrix}}$ | $1$ | $\frac{1}{2}$ | $0$ | $-\frac{1}{2}$ | $0$ | $-\frac{1}{2}r$ | $\frac{1}{4}(1-2r)$ | $-\frac{1}{4}(1-2r)$ | $-\frac{1}{4}(1-2r)$ |  | $1$ | $\frac{1}{2}$ | $\frac{1}{2}$ | $-\frac{1}{2}$ | $\frac{1}{2}$ | $-\frac{1}{2}r$ | $\frac{1}{2}(1-r)$ | $-\frac{1}{2}(1-r)$ | $\frac{1}{2}r$ |
| $\frac{\begin{matrix} A & B \end{matrix}}{\begin{matrix} a & B \end{matrix}}$ | $1$ | $0$ | $0$ | $\frac{1}{2}$ | $0$ | $\frac{1}{4}(1-2r)$ | $-\frac{1}{4}(1-2r)$ | $0$ | $0$ |  | $1$ | $0$ | $\frac{1}{2}$ | $\frac{1}{2}$ | $\frac{1}{2}$ | $\frac{1}{4}(1-2r)$ | $-\frac{1}{4}(1-2r)$ | $\frac{1}{4}$ | $\frac{1}{4}$ |
| $\frac{\begin{matrix} A & B \end{matrix}}{\begin{matrix} a & b \end{matrix}}$ | $1$ | $0$ | $0$ | $0$ | $0$ | $\frac{1}{2}(1-2r)$ | $0$ | $0$ | $\frac{1}{4}(1-2r)^{2}$ |  | $1$ | $0$ | $\frac{1}{2}$ | $0$ | $\frac{1}{2}$ | $\frac{1}{2}(1-2r)$ | $0$ | $0$ | $\frac{1}{2}[r^{2}+(1-r)^{2}]$ |
| $\frac{\begin{matrix} A & b \end{matrix}}{\begin{matrix} a & B \end{matrix}}$ | $1$ | $0$ | $0$ | $0$ | $0$ | $0$ | $0$ | $0$ | $-\frac{1}{4}(1-2r)^{2}$ |  | $1$ | $0$ | $\frac{1}{2}$ | $0$ | $\frac{1}{2}$ | $0$ | $0$ | $0$ | $r(1-r)$ |
| $\frac{\begin{matrix} A & b \end{matrix}}{\begin{matrix} a & b \end{matrix}}$ | $1$ | $0$ | $0$ | $-\frac{1}{2}$ | $0$ | $\frac{1}{4}(1-2r)$ | $\frac{1}{4}(1-2r)$ | $0$ | $0$ |  | $1$ | $0$ | $\frac{1}{2}$ | $-\frac{1}{2}$ | $\frac{1}{2}$ | $\frac{1}{4}(1-2r)$ | $\frac{1}{4}(1-2r)$ | $-\frac{1}{4}$ | $\frac{1}{4}$ |
| $\frac{\begin{matrix} a & B \end{matrix}}{\begin{matrix} a & B \end{matrix}}$ | $1$ | $-\frac{1}{2}$ | $0$ | $\frac{1}{2}$ | $0$ | $-\frac{1}{2}r$ | $-\frac{1}{4}(1-2r)$ | $\frac{1}{4}(1-2r)$ | $-\frac{1}{4}(1-2r)$ |  | $1$ | $-\frac{1}{2}$ | $\frac{1}{2}$ | $\frac{1}{2}$ | $\frac{1}{2}$ | $-\frac{1}{2}r$ | $-\frac{1}{2}(1-r)$ | $\frac{1}{2}(1-r)$ | $\frac{1}{2}r$ |
| $\frac{\begin{matrix} a & B \end{matrix}}{\begin{matrix} a & b \end{matrix}}$ | $1$ | $-\frac{1}{2}$ | $0$ | $0$ | $0$ | $\frac{1}{4}(1-2r)$ | $0$ | $\frac{1}{4}(1-2r)$ | $0$ |  | $1$ | $-\frac{1}{2}$ | $\frac{1}{2}$ | $0$ | $\frac{1}{2}$ | $\frac{1}{4}(1-2r)$ | $-\frac{1}{4}$ | $\frac{1}{4}(1-2r)$ | $\frac{1}{4}$ |
| $\frac{\begin{matrix} a & b \end{matrix}}{\begin{matrix} a & b \end{matrix}}$ | $1$ | $-\frac{1}{2}$ | $0$ | $-\frac{1}{2}$ |  |  |  |  |  |  |  |  |  |  |  |  |  |  |  |
