# Supplementary material for: A Complete Solution for Dissecting Pure Main and Epistatic Effects of QTL in Triple Testcross Design
Source: PLoS One. 2011 Sep 19;6(9):e24575. doi: 10.1371/journal.pone.0024575 (PMC3176238; doi:10.1371/journal.pone.0024575)
Supplement: Table S7 — Expected genetic values of ,and under the F2 metric model in the RIL-based TTC design. (DOC) [file pone.0024575.s010.doc]

**Table S7**

**Expected genetic values of $Z_{1i}=\bar{L}_{1i}+\bar{L}_{2i}$,$Z_{2i}=\bar{L}_{1i}-\bar{L}_{2i}$and $Z_{3i}=\bar{L}_{1i}+\bar{L}_{2i}-2\bar{L}_{3i}$ under the F_2_ metric model**

**in the RIL-based** TTC design

| Genotype of  RIL plant | Frequency | $Z_{1i}$ | | | | | | | | |  | $Z_{2i}$ | | | | | | | |  | $Z_{3i}$ | | | |
| --- | --- | --- | --- | --- | --- | --- | --- | --- | --- | --- | --- | --- | --- | --- | --- | --- | --- | --- | --- | --- | --- | --- | --- | --- |
|  |  | $\mu$ | $a_{1}$ | $d_{1}$ | $a_{2}$ | $d_{2}$ | $i_{a_{1}a_{2}}$ | $i_{a_{1}d_{2}}$ | $i_{d_{1}a_{2}}$ | $i_{d_{1}d_{2}}$ |  | $a_{1}$ | $d_{1}$ | $a_{2}$ | $d_{2}$ | $i_{a_{1}a_{2}}$ | $i_{a_{1}d_{2}}$ | $i_{d_{1}a_{2}}$ | $i_{d_{1}d_{2}}$ |  | $i_{a_{1}a_{2}}$ | $i_{a_{1}d_{2}}$ | $i_{d_{1}a_{2}}$ | $i_{d_{1}d_{2}}$ |
| $\frac{\begin{matrix} A & B \end{matrix}}{\begin{matrix} A & B \end{matrix}}$ | $\frac{1}{2}(1-R)$ | $2$ | $1$ | $0$ | $1$ | $0$ | $1$ | $-\frac{1}{2}$ | $-\frac{1}{2}$ | $\frac{1}{2}$ |  | $1$ | $-1$ | $1$ | $-1$ | $1$ | $-\frac{1}{2}$ | $-\frac{1}{2}$ | $0$ |  | $r$ | $-r$ | $-r$ | $r$ |
| $\frac{\begin{matrix} A & b \end{matrix}}{\begin{matrix} A & b \end{matrix}}$ | $\frac{1}{2}R$ | $2$ | $1$ | $0$ | $-1$ | $0$ | $0$ | $\frac{1}{2}$ | $-\frac{1}{2}$ | $-\frac{1}{2}$ |  | $1$ | $-1$ | $1$ | $1$ | $0$ | $\frac{1}{2}$ | $\frac{1}{2}$ | $0$ |  | $r$ | $r$ | $-r$ | $-r$ |
| $\frac{\begin{matrix} a & B \end{matrix}}{\begin{matrix} a & B \end{matrix}}$ | $\frac{1}{2}R$ | $2$ | $-1$ | $0$ | $1$ | $0$ | $0$ | $-\frac{1}{2}$ | $\frac{1}{2}$ | $-\frac{1}{2}$ |  | $1$ | $1$ | $1$ | $-1$ | $0$ | $\frac{1}{2}$ | $\frac{1}{2}$ | $0$ |  | $r$ | $-r$ | $r$ | $-r$ |
| $\frac{\begin{matrix} a & b \end{matrix}}{\begin{matrix} a & b \end{matrix}}$ | $\frac{1}{2}(1-R)$ | $2$ | $-1$ | $0$ | $-1$ | $0$ | $1$ | $\frac{1}{2}$ | $\frac{1}{2}$ | $\frac{1}{2}$ |  | $1$ | $1$ | $1$ | $1$ | $-1$ | $-\frac{1}{2}$ | $-\frac{1}{2}$ | $0$ |  | $r$ | $r$ | $r$ | $r$ |

*$R=2r/(1+2r)$.
